# O-GlcNAcylation regulates microglial neuroinflammation in Parkinson’s disease

**DOI:** 10.1038/s41531-026-01319-6

**Published:** 2026-03-28

**Authors:** Dong Yeol Kim, Sang-Min Kim, Chanhaeng Lee, Inn-Oc Han

**Affiliations:** Department of Physiology and Biophysics, College of Medicine, Inha University, Incheon, Korea. Department of Biomedical Science, Program in Biomedical Science and Engineering, Inha University, Incheon, Korea

**Keywords:** Diseases, Immunology, Neurology, Neuroscience

## Abstract

*O*-GlcNAcylation, a nutrient-sensitive post-translational modification, has emerged as a key regulator of immune and inflammatory processes. However, its role in neuroinflammation and neurodegenerative disease progression remains poorly defined. In this study, we explored how reduced *O*-GlcNAcylation contributes to neuroinflammatory signaling in Parkinson’s disease (PD), a disorder increasingly recognized to involve dysregulated immune–metabolic interactions. Analysis of postmortem PD substantia nigra (SN) revealed a marked reduction in global *O*-GlcNAcylation levels, concomitant with enhanced neuroinflammatory signatures and a predominance of pro-inflammatory microglial activation states. In a lipopolysaccharide (LPS)-induced PD mouse model, pharmacological elevation of *O*-GlcNAcylation through glucosamine (GlcN) or the Thiamet-G significantly ameliorated motor deficits, preserved tyrosine hydroxylase (TH)-positive dopaminergic neurons, and attenuated neuroinflammatory responses, including glial activation and inflammasome assembly. In primary microglial cultures, enhanced *O*-GlcNAcylation suppressed LPS-induced pro-inflammatory gene expression while promoting anti-inflammatory and homeostatic phenotypes. Mechanistically, increased *O*-GlcNAcylation dampened NF-κB signaling activity and reduced the production of pro-inflammatory cytokines, thereby reprogramming microglial functional states. Collectively, these findings identify *O*-GlcNAcylation as a critical modulator of microglial-mediated neuroinflammation and highlight its therapeutic potential for inflammation-associated neurodegenerative disorders such as PD.

## Introduction

PD is a progressive neurodegenerative disorder that primarily affects dopaminergic neurons in the substantia nigra pars compacta and is characterized by motor symptoms such as bradykinesia, rigidity, resting tremor, and postural instability^1^. While the hallmark pathological features include dopaminergic neuronal loss and accumulation of α-synuclein aggregates, the underlying mechanisms of neurodegeneration are multifactorial, involving oxidative stress, mitochondrial dysfunction, autophagy impairment, endoplasmic reticulum stress, and, notably, chronic neuroinflammation^1–3^.

Neuroinflammation has emerged as a central pathological mechanism contributing to the progression of PD^4^. Among the key mediators of neuroinflammation, microglia—the principal resident immune cells of the central nervous system (CNS)—play a pivotal role by responding to pathological stimuli and orchestrating inflammatory cascades. Activated microglia release pro-inflammatory cytokines, reactive oxygen species, and engage inflammasome signaling pathways, which can exacerbate neuronal injury and synaptic dysfunction^4,5^. Microglia exhibit remarkable phenotypic plasticity, shifting between a surveillant (resting) state and activated states in response to environmental and pathological cues^6,7^. Microglial activation encompasses a highly dynamic and context-dependent continuum that integrates both neurotoxic and neuroprotective functions^6,7^. Depending on the nature, intensity, and duration of stimuli, microglia may either promote inflammation and neuronal damage or, conversely, facilitate the clearance of cellular debris and misfolded proteins while supporting tissue repair and neuronal survival^8,9^. The ability of microglia to maintain a balanced transition across these functional states is essential for preserving CNS homeostasis^9,10^. In PD, chronic and maladaptive microglial activation—characterized by sustained pro-inflammatory signaling and impaired resolution mechanisms—has been closely linked to dopaminergic neurodegeneration in the SN and the progression of motor deficits^10,11^.

To investigate inflammation-mediated pathogenesis in PD, the LPS-induced model has been widely utilized in both in vivo and in vitro experimental systems^12–14^. In the in vivo model, stereotaxic injection of LPS into the SN of rodents triggers localized microglial activation and a robust neuroinflammatory response, effectively recapitulating key features of inflammation-driven dopaminergic neurodegeneration^15^. This targeted approach specifically activates microglial Toll-like receptor 4 (TLR4) signaling within the SN, leading to the upregulation of NF-κB-dependent pro-inflammatory cytokines^16^. The resulting inflammatory cascade promotes the degeneration of dopaminergic neurons in the SN, reproducing core pathological features of PD with high anatomical and mechanistic fidelity. Notably, LPS-induced neuroinflammation drives microglia toward a persistently activated, pro-inflammatory, and neurotoxic state, characterized by elevated production of cytokines, chemokines, and oxidative mediators that exacerbate neuronal injury and disease progression^17–19^. This sustained activation further amplifies the inflammatory milieu, creating a self-perpetuating cycle of neuroinflammation and neuronal damage^20,21^. In vitro, stimulation of microglial cells with LPS similarly induces a pronounced activation response characterized by upregulation of inflammatory signaling pathways and effector molecules^22,23^. This in vitro model provides a reproducible and mechanistically tractable platform for elucidating the molecular determinants of microglial activation and inflammation-associated neurotoxicity.

*O*-GlcNAcylation is a nutrient-sensitive, dynamic post-translational modification in which *O*-linked N-acetylglucosamine (*O*-GlcNAc) is attached to serine or threonine residues of nuclear and cytoplasmic proteins by *O*-GlcNAc transferase (OGT) and removed by *O*-GlcNAcase (OGA)^24,25^. By sensing and integrating cellular metabolic fluxes, *O*-GlcNAcylation modulates a broad spectrum of cellular functions, including transcriptional regulation, signal transduction, proteostasis, and adaptive stress responses^26–28^. Recent studies have highlighted a critical role for *O*-GlcNAcylation in modulating inflammatory signaling pathways, including inhibition of NF-κB activation and reduction of cytokine production in both peripheral and central systems^29,30^. In the context of neurodegeneration, our previous studies have demonstrated that pharmacologically increasing *O*-GlcNAcylation confers neuroprotection in rodent models of ischemic stroke and toxin-induced Parkinsonism^31–33^. These protective effects were linked to suppression of inflammatory responses and preservation of mitochondrial integrity. However, whether *O*-GlcNAcylation directly regulates microglial activation and thereby modulates neuroinflammation in PD has remained unclear.

In this study, we investigated the regulatory role of *O*-GlcNAcylation in microglial activation and neuroinflammatory signaling within PD models. Given its metabolic sensitivity, *O*-GlcNAcylation may function as a critical link between cellular metabolism and neuroinflammatory responses in neurodegeneration.

## Results

### Reduced O-GlcNAcylation accompanies neuroinflammation and pro-inflammatory microglial activation in the SN of PD patients

**Fig. 1:**
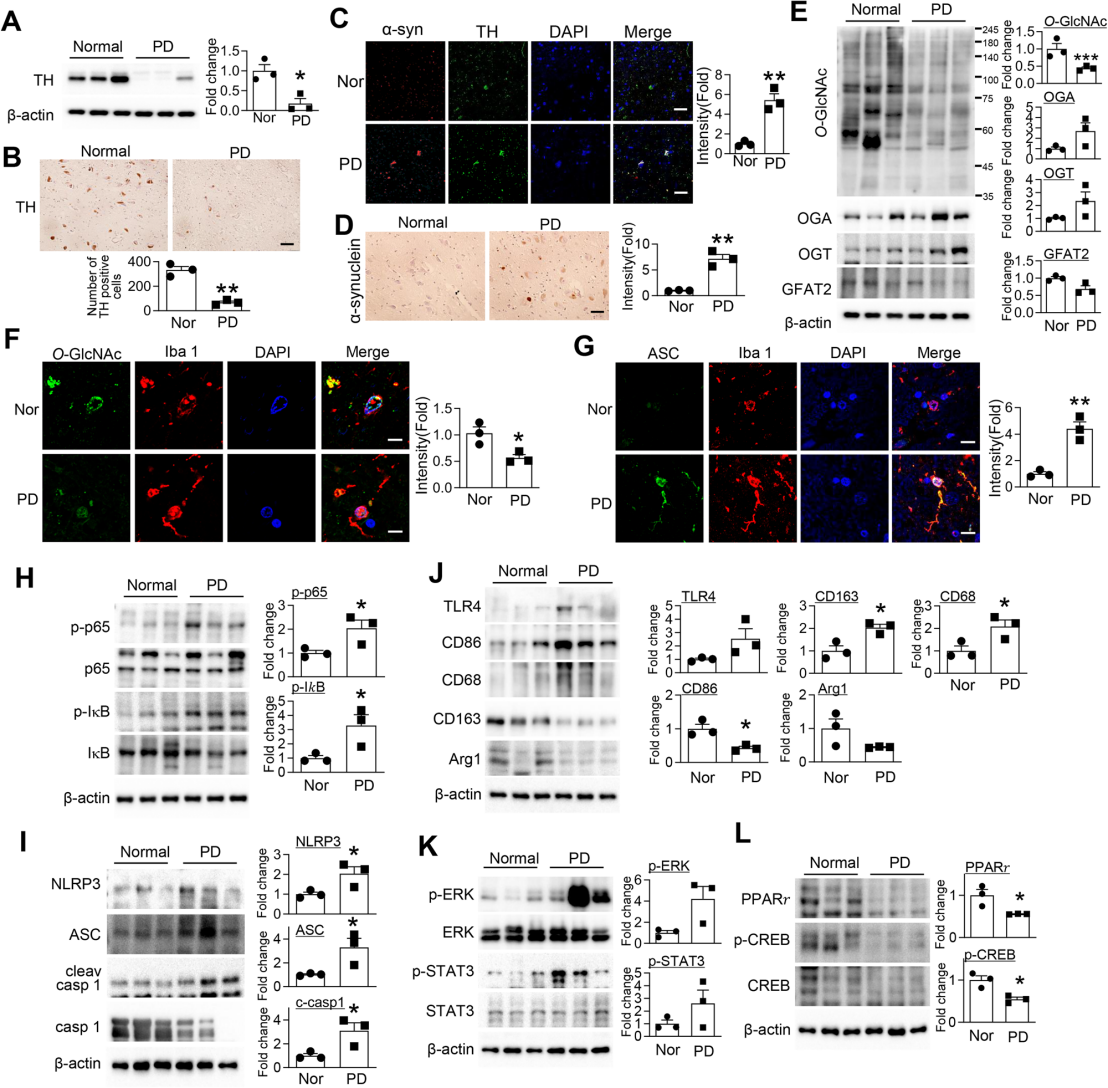
Reduced *O*-GlcNAcylation accompanies neuroinflammation and pro-inflammatory microglial activation in the SN of PD patients. **A** Western blot analysis of midbrain tissue lysates was performed using TH and β-actin antibodies. Quantification of TH expression was normalized to β-actin (*n* = 3 per group). **B** Representative immunohistochemical images of TH in human midbrain sections. Scale bar = 50 μm (*n* = 3 per group). **C** Co-immunostaining of α-synuclein with TH-positive neurons in the human midbrain. Representative merged images are shown (*n* = 3 per group). **D** Immunostaining of α-synuclein in midbrain sections. Scale bar = 50 μm (*n* = 3 per group). **E** Western blot analysis of *O*-GlcNAcylation levels and the expression of OGT, OGA, GFAT2 and β-actin in midbrain lysates. Quantification was normalized to β-actin (*n* = 3 per group). **F** Representative immunofluorescent images of primary microglial cells stained for *O*-GlcNAc (green), Iba1 (red), and DAPI (blue). Scale bar = 10 μm (*n* = 3 per group). **G** Representative immunofluorescent images of primary microglial cells stained for ASC (green), Iba1 (red), and DAPI (blue). Scale bar = 10 μm (*n* = 3 per group). **H** Western blot analysis of NF-κB signaling components in midbrain lysates, including phospho-p65 (p-p65), total p65, phospho-IκB (p-IκB), total IκB, and β-actin. Quantification of p-p65 was normalized to p65; p-IκB was normalized to IκB (*n* = 3 per group). **I** Western blot analysis of neuroinflammatory markers in midbrain lysates, including TLR4, CD86, CD68, CD163, Arg1, and β-actin. Quantification was normalized to β-actin (*n* = 3 per group). **J** Western blot analysis of inflammasome-related proteins in midbrain lysates, including NLRP3, ASC, cleaved caspase-1, total caspase-1, and β-actin. Quantification of NLRP3 and ASC was normalized to β-actin; cleaved caspase-1 was normalized to total caspase-1 (*n* = 3 per group). **K** Western blot analysis of phosphorylated and total STAT3 and ERK in midbrain lysates. Quantification of p-STAT3 was normalized to STAT3; p-ERK was normalized to ERK (*n* = 3 per group). **L** Western blot analysis of PPARγ, phosphorylated CREB (p-CREB), total CREB, and β-actin in midbrain lysates. Quantification of p-CREB was normalized to total CREB; PPARγ was normalized to β-actin (*n* = 3 per group). Data are presented as mean SEM; ^*^p < 0.05, ^**^p < 0.01, ^***^p < 0.001. Statistical analysis was performed using Student *t* test.

In the SN of postmortem brains from PD patients, we observed several pathological alterations consistent with neurodegeneration, including a significant reduction in TH-positive neurons and accumulation of α-synuclein (Fig. 1A–D). Alongside these changes, global *O*-GlcNAcylation levels were notably decreased in PD brain tissue compared to age-matched controls (Fig. 1E). Although OGT and OGA showed a trend toward increased expression in PD samples, these changes were not statistically significant. GFAT2 expression was also not significantly different between PD patients and controls. Immunofluorescence analysis further revealed that microglia within the PD SN exhibited lower *O*-GlcNAcylation, highlighting a distinct microglia-associated alteration in the diseased environment (Fig. 1F). Furthermore, PD patients displayed increased apoptosis-associated speck-like protein containing a CARD (ASC) expression in microglia, indicating increased inflammasome activation (Fig. 1G). In parallel, markers of neuroinflammation were elevated in the PD brain, as evidenced by enhanced NF-κB signaling, including increased phosphorylation of p65 and IκB (Fig. 1H) and upregulation of NLRP3, ASC, and cleaved caspase-1, indicating inflammasome activation (Fig. 1I). Microglial phenotyping revealed a shift toward a pro-inflammatory and activated state, as indicated by increased expression of TLR4, CD86, and CD68, accompanied by a reduction in resting-state marker CD163 (Fig. 1J). Furthermore, inflammatory signaling pathways, including phosphorylated ERK and STAT3, were markedly upregulated, whereas anti-inflammatory regulators such as PPARγ and phosphorylated CREB were diminished in the brains of PD patients (Fig. 1K, L). These findings indicate that reduced *O*-GlcNAcylation is associated with enhanced inflammatory signaling and dysregulated microglial activation in the PD brain, suggesting a potential link between disrupted protein *O*-GlcNAcylation and neuroimmune dysregulation in PD.

### Augmenting O-GlcNAcylation with GlcN attenuates LPS-induced neuroinflammation and dopaminergic neurodegeneration in a murine PD model

**Fig. 2:**
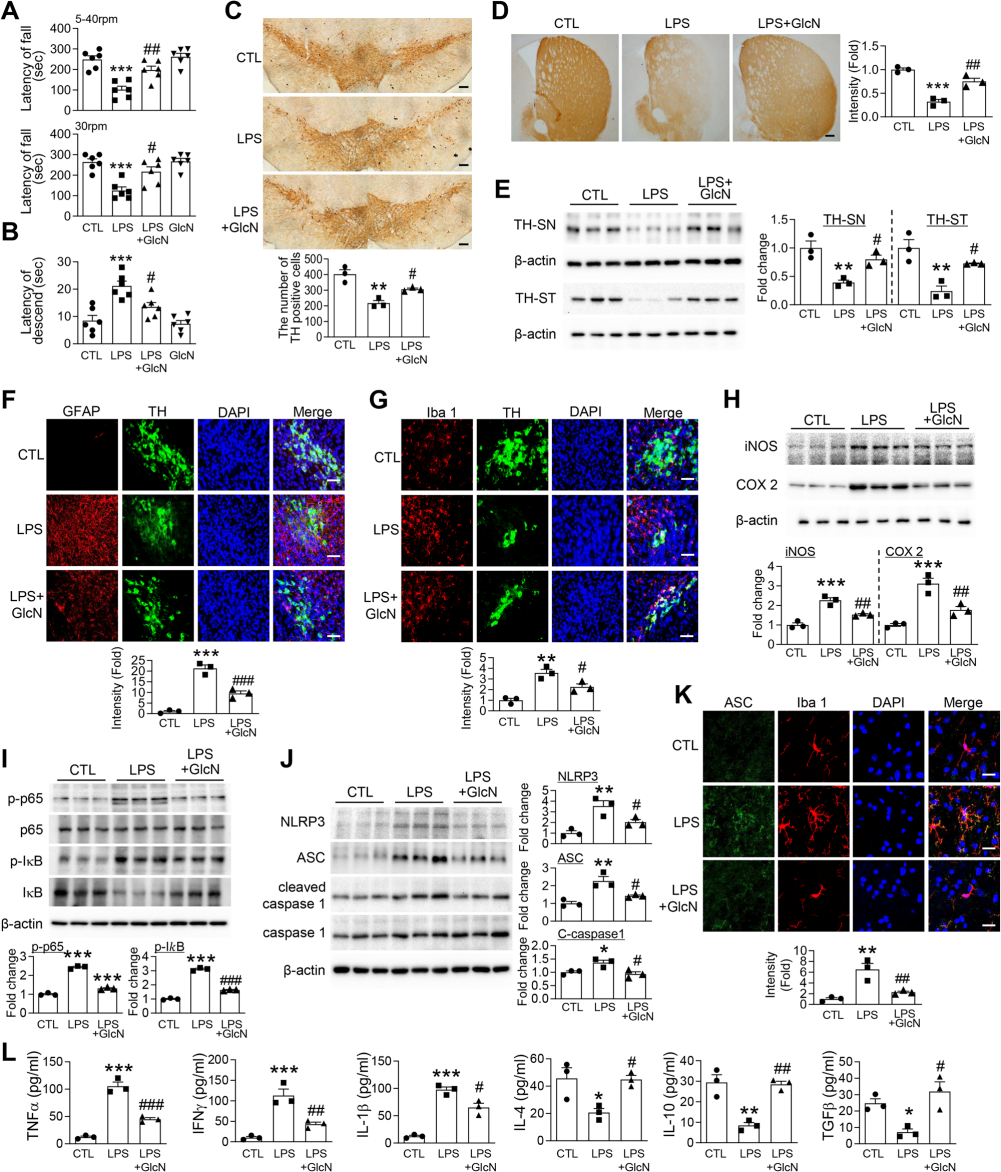
Augmenting *O*-GlcNAcylation with GlcN attenuates LPS-induced neuroinflammation and dopaminergic neurodegeneration in a murine PD model. The mice received bilateral injections of LPS (5 μg) into the SN of the brain. GlcN was administered intraperitoneally at a dose of 200 mg/kg, three times per week for 4 weeks. Motor coordination and bradykinesia were evaluated using the rotarod test (**A**) and pole test (**B**) at 4 weeks post-injection (*n* = 6 per group). TH immunohistochemistry was performed to assess dopaminergic neurons in the SN (**C**) and ST (**D**). Scale bars = 100 μm (*n* = 3 per group). (E) Western blot analysis of TH expression in SN lysates. Quantification was normalized to β-actin (*n* = 3 per group). Representative immunofluorescence images of SN tissue showing GFAP (red), TH (green), DAPI (blue) (**F**) and Iba1 (red), TH (green), DAPI (blue) (**G**). Scale bars = 50 μm (*n* = 3 per group). **H** Western blot analysis of inflammatory markers iNOS and COX2 in SN lysates. Quantification was normalized to β-actin (*n* = 3 per group). **I** Western blot analysis of NF-κB pathway components, including phospho-p65 (p-p65), phospho-IκB (p-IκB), total p65, and total IκB in SN lysates. Quantification of p-p65 was normalized to total p65, and p-IκB was normalized to total IκB (*n* = 3 per group). **J** Western blot analysis of inflammasome-related proteins, including NLRP3, ASC, cleaved caspase-1, total caspase-1, and β-actin in SN lysates. Quantification of NLRP3 and ASC was normalized to β-actin; cleaved caspase-1 was normalized to total caspase-1 (*n* = 3 per group). **K** Representative immunofluorescence images of ASC (green), Iba1 (red), and DAPI (blue) in SN tissue. Scale bar = 10 μm (*n* = 3 per group). **L** ELISA analysis of pro-inflammatory cytokines (TNF-α, IFN-γ, IL-1β) and anti-inflammatory cytokines (IL-4, IL-10, TGF-β) in SN tissue (*n* = 3 per group). Data are presented as mean SEM; ^*^p < 0.05, ^**^p < 0.01, ^***^p < 0.001 versus control, ^#^*p* < 0.05, ^##^*p* < 0.01, ^###^*p* < 0.001 versus LPS. Statistical analysis was performed using one-way ANOVA with Tukey’s post hoc multiple comparison test.

Given emerging reports that *O*-GlcNAc cycling plays a regulatory role in neuroinflammation, we sought to investigate this relationship in the context of PD by evaluating whether GlcN-mediated enhancement of *O*-GlcNAcylation could mitigate LPS-induced PD pathology in a mouse model. To induce neuroinflammation and dopaminergic neurodegeneration, mice received stereotaxic injections of LPS into the SN, with a subset concurrently treated with GlcN. LPS-injected mice exhibited significant motor impairments, as evidenced by decreased latency to fall in the rotarod test (Fig. 2A) and increased descent time in the pole test (Fig. 2B). Notably, these deficits were fully reversed by GlcN co-administration. Immunohistochemical analysis revealed that LPS markedly decreased TH–positive neuron counts in the SN (Fig. 2C) and TH-positive fiber density in the striatum (ST; Fig. 2D), findings corroborated by regional TH protein reductions on Western blot (Fig. 2E); GlcN reversed these dopaminergic losses. Concurrently, LPS provoked pronounced glial activation, as evidenced by elevated GFAP and Iba1 immunoreactivity (Fig. 2F, G), upregulation of COX-2, iNOS, phosphorylated p65 and IκB (Fig. 2H–I), and increased inflammasome markers (Fig. 2J, K); all neuroinflammatory indices were significantly attenuated by GlcN. In parallel, LPS increased oxidative stress in the SN, as indicated by elevated H₂O₂ levels and increased NOX2 protein expression, both of which were significantly attenuated by GlcN treatment. (Fig. S1A, S1B). Finally, LPS exposure altered the cytokine profile by promoting a pro-inflammatory shift—marked by increased levels of TNF-α, IFN-γ, and IL-1β—and a concomitant reduction in anti-inflammatory cytokines such as IL-4, IL-10, and TGF-β. This imbalance was effectively restored by GlcN treatment (Fig. 2L). To assess whether GlcN-mediated regulation of neuroinflammation involves transcriptional control, we analyzed mRNA expression of key inflammatory mediators. LPS administration increased transcripts of inflammasome components (NLRP3 and ASC) and pro-inflammatory cytokines (TNF-α, IFN-γ, and IL-1β), while concomitantly reducing anti-inflammatory cytokine genes (IL-4, IL-10, and TGF-β). Co-administration of GlcN significantly reversed these transcriptional changes (Fig. S1C, S1D), indicating that enhancement of *O*-GlcNAcylation modulates the inflammatory milieu, at least in part, at the transcriptional level. Collectively, our findings demonstrate that augmenting O-GlcNAcylation effectively counteracts LPS-induced motor dysfunction, dopaminergic neuron loss, and neuroinflammation in this mouse model.

### Increased O-GlcNAcylation by Thiamet G restores motor function, dopaminergic integrity, and attenuates neuroinflammation in an LPS-induced PD mouse model

**Fig. 3:**
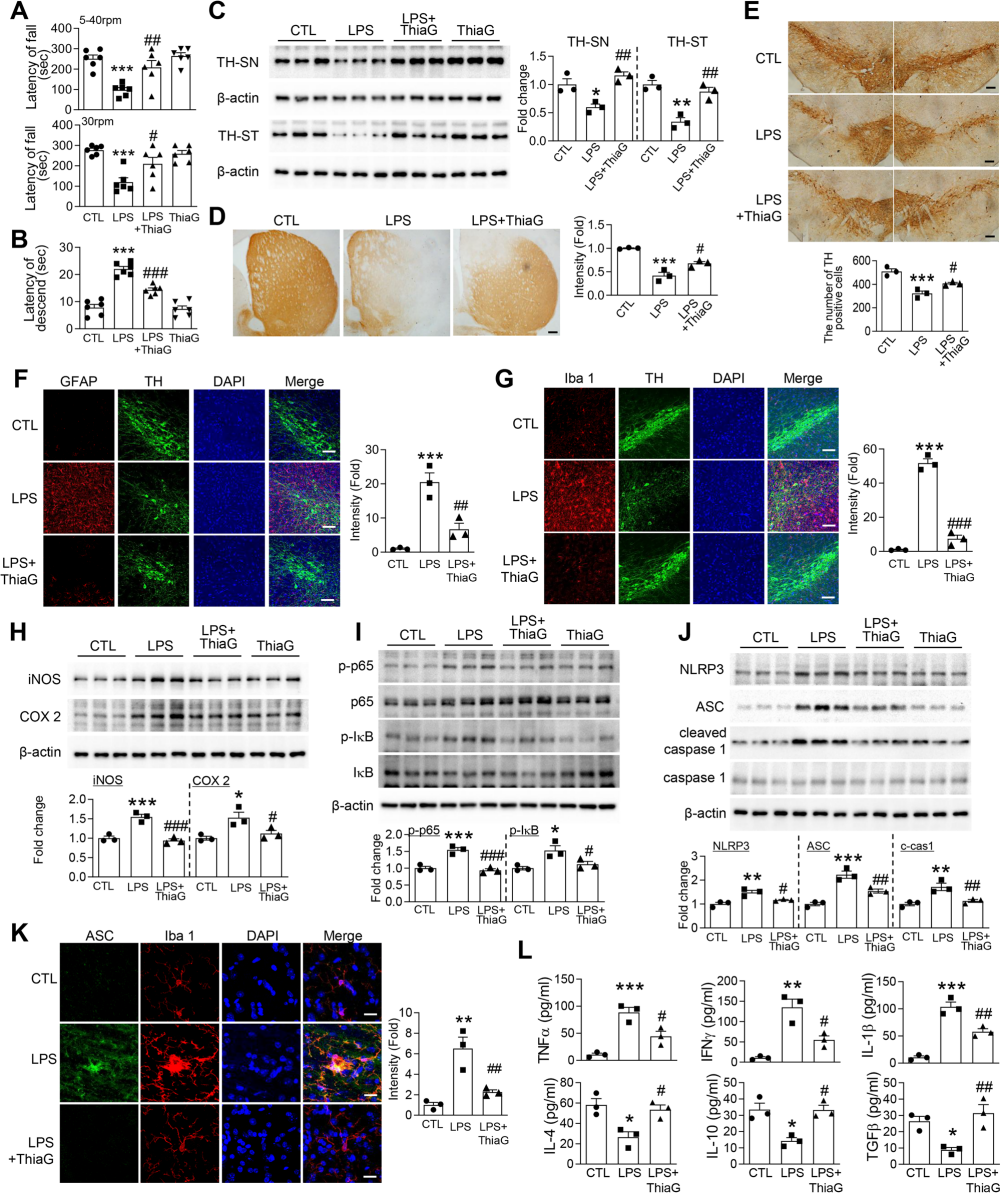
Inhibition of *O*-GlcNAcase by Thiamet G restores motor function, dopaminergic integrity, and attenuates neuroinflammation in an LPS-induced PD mouse model. The mice received bilateral injections of LPS (5 μg) into the SN of the brain to induce neuroinflammation. Thiamet G (ThiaG) was administered intraperitoneally at a dose of 20 mg/kg, three times per week for four weeks. Motor coordination and bradykinesia were assessed using the rotarod test (**A**) and pole test (**B**) at 4 weeks post-injection (*n* = 6 per group). **C** Western blot analysis of TH expression in SN tissue lysates. TH levels were normalized to β-actin (*n* = 3 per group). Dopaminergic neuronal loss was visualized via TH immunohistochemistry in the ST (**D**) and SN (**E**). Scale bars = 100 μm (*n* = 3 per group). Representative immunofluorescence images of SN sections stained for TH (green), GFAP (astrocyte marker, red; **F**), Iba1 (microglial marker, red; **G**), and DAPI (nuclear counterstain, blue). Scale bars = 50 μm (*n* = 3 per group). **H** Western blot analysis of pro-inflammatory enzymes iNOS and COX2 in SN tissue lysates. Expression levels were normalized to β-actin (*n* = 3 per group). **I** Western blot analysis of NF-κB signaling pathway proteins, including phosphorylated and total p65 and IκB. The levels of p-p65 and p-IκB were normalized to total p65 and IκB, respectively (*n* = 3 per group). **J** Western blot analysis of inflammasome components, including NLRP3, ASC, cleaved caspase-1, and total caspase-1. NLRP3 and ASC levels were normalized to β-actin, while cleaved caspase-1 was normalized to total caspase-1 (*n* = 3 per group). **K** Representative immunofluorescence images of SN tissue showing ASC (green), Iba1 (red), and DAPI (blue). Scale bar = 10 μm (*n* = 3 per group). **L** ELISA analysis of pro-inflammatory cytokines (TNF-α, IFN-γ, IL-1β) and anti-inflammatory cytokines (IL-4, IL-10, TGF-β) in SN tissue lysates (*n* = 3 per group). Data are presented as mean SEM; ^**^p < 0.05, ^**^p < 0.01, ^***^p < 0.001 versus control, ^#^*p* < 0.05, ^##^*p* < 0.01, ^###^*p* < 0.001 versus LPS. Statistical analysis was performed using one-way ANOVA with Tukey’s post hoc multiple comparison test.

We next assessed whether pharmacological inhibition of *O*-GlcNAcase with Thiamet G similarly mitigates LPS-induced pathology. Mice received stereotaxic LPS infusions into the SN followed by systemic Thiamet G treatment, which restored motor function in both the rotarod (Fig. 3A) and pole tests (Fig. 3B) to levels indistinguishable from controls. Immunohistochemical and Western blot analyses demonstrated that Thiamet G significantly rescued the LPS-driven loss of TH-positive neurons in the SN and TH-positive axonal projections in the ST (Fig. 3C–E). Consistent with its neuroprotective effects, Thiamet G markedly attenuated LPS-induced astrogliosis and microgliosis (Fig. 3F, G). In parallel, Thiamet G significantly reduced oxidative stress, as evidenced by decreased H₂O₂ levels and NOX2 protein expression, and suppressed the induction of COX-2, iNOS, phosphorylated p65, and IκB (Figs. 3H, I, S1E, S1F). Furthermore, inflammasome activation was attenuated, as indicated by reduced cleaved caspase-1 levels and diminished ASC speck formation (Fig. 3J). Notably, LPS treatment induced robust ASC expression specifically within Iba1-positive microglia, indicating microglial inflammasome assembly (Fig. 3K). Finally, multiplex cytokine analysis demonstrated that Thiamet G effectively reversed the LPS-induced shift toward pro-inflammatory milieu, including TNF-α, IFN-γ, and IL-1β, while restoring anti-inflammatory cytokines such as IL-4, IL-10, and TGF-β (Fig. 3L). To determine whether this immunomodulatory effect occurs at the transcriptional level, we assessed mRNA expression of key inflammatory mediators. LPS administration markedly increased transcripts encoding inflammasome components (NLRP3 and ASC) and pro-inflammatory cytokines (TNF-α, IFN-γ, and IL-1β), while concomitantly suppressing anti-inflammatory cytokine genes (IL-4, IL-10, and TGF-β). These transcriptional alterations were significantly reversed by Thiamet G co-administration (Fig. S1G, S1H), indicating that enhancement of *O*-GlcNAcylation modulates the inflammatory milieu, at least in part, through transcriptional regulation. Together, these data reinforce the pivotal role of *O*-GlcNAc cycing in controlling neuroinflammation and dopaminergic degeneration in LPS models.

### GlcN and Thiamet G counteract LPS-induced decrease of microglial O-GlcNAcylation in the SN

**Fig. 4:**
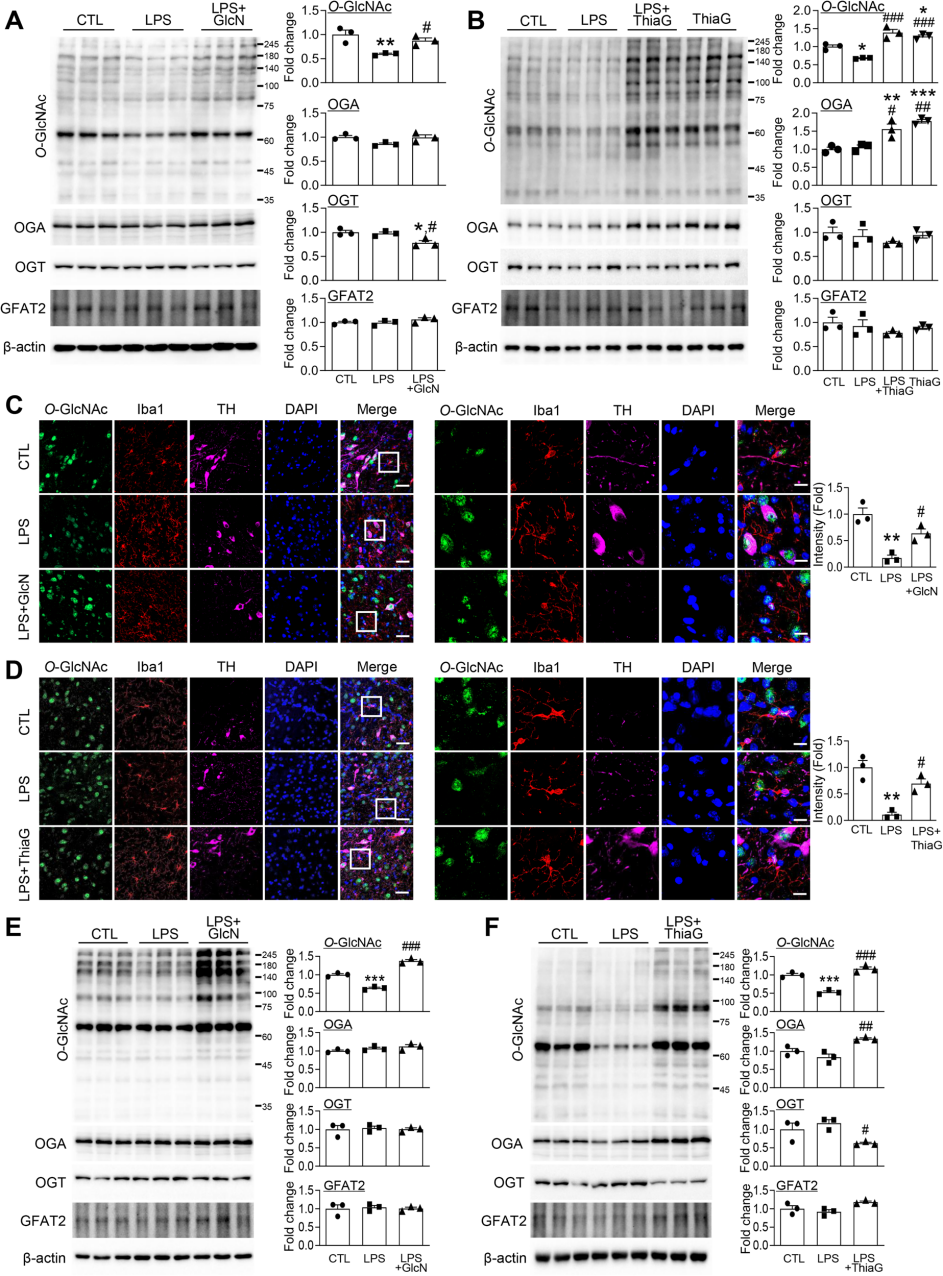
GlcN and Thiamet G counteract LPS-induced decrease of microglial *O*-GlcNAcylation in the SN. LPS (5 μg) was injected into the SN region of mouse brain. Some groups received intraperitoneal injections of GlcN (200 mg/kg) (**A**, **C**) or Thiamet G (20 mg/kg) (**B**, **D**) three times a week for 4weeks. **A**, **B** Western blot analysis of SN tissue lysates was performed using *O*-GlcNAc, OGT, OGA, GFAT2, and β-actin antibodies. *O*-GlcNAc, OGT, OGA, and GFAT2 quantification was normalized to β-actin (n = 3/group). **C**, **D** Representative immunofluorescent images for *O*-GlcNAc (green), Iba1 (red), TH (violet), DAPI (blue), and a merged image of SN in mouse brains. Scale bar represents 50 μm (*n* = 3/group). Primary microglial cells treated with LPS (400 ng/ml) with or without GlcN (5 mM) (E) or Thiamet G (1μM) (**F**) for 24 h. Western blot analysis of primary microglia cell lysates was performed using *O*-GlcNAc, OGT, OGA, GFAT2, and β-actin antibodies. *O*-GlcNAc, OGA, OGT, and GFAT2 quantification was normalized to β-actin (n = 3/group). Data are presented as mean SEM; ^*^p < 0.05, ^**^p < 0.01, ^***^p < 0.001 versus control, ^#^*p* < 0.05, ^##^*p* < 0.01, ^###^*p* < 0.001 versus LPS. Statistical analysis was performed using one-way ANOVA with Tukey’s post hoc multiple comparison test.

In line with our prior observation that diverse inflammatory stimuli diminish *O*-GlcNAc levels^31–33^, stereotaxic LPS injection into the mouse SN resulted in a significant reduction of global *O*-GlcNAcylation (Fig. 4A, B). Systemic administration of either GlcN or Thiamet G fully reinstated total *O*-GlcNAc levels in the SN (Fig. 4A, B). Importantly, immunofluorescence staining revealed that this LPS-induced depletion was pronounced within Iba1-positive microglia and was likewise reversed by both GlcN and Thiamet G treatment (Fig. 4C, D). To investigate this effect at the cellular level, primary microglia were cultured and treated with LPS in the presence or absence of GlcN or Thiamet G. Western blot analysis confirmed that LPS significantly reduced *O*-GlcNAc levels in microglia, whereas co-treatment with GlcN or Thiamet G restored *O*-GlcNAc expression to baseline levels (Fig. 4E, F). Notably, Thiamet G treatment increased OGA protein expression both in the SN and in primary microglia (Fig. 4B, F), while GlcN did not significantly alter OGA levels (Fig. 4A, E). Consistent with these findings, Thiamet G significantly upregulated *Oga* mRNA expression in both primary microglia and SN tissue (Fig. S2A, S2B). To determine whether these changes in OGA expression were accompanied by altered enzymatic activity, we measured OGA activity in mouse brain lysates. LPS treatment did not significantly alter OGA activity compared with control, and GlcN had no effect. Thiamet G, however, significantly reduced OGA activity (Fig. S3A, S3B). By comparison, expression of GFAT2 was not altered by LPS exposure or by GlcN or Thiamet G treatment in either the SN or primary microglia (Fig. 4A, B, E, F). Collectively, these data indicate that both GlcN and Thiamet G counteract inflammation-associated reductions in *O*-GlcNAcylation in microglia, in vivo and in vitro.

### Restoration of O-GlcNAcylation in Microglia Suppresses LPS-induced inflammatory and inflammasome signaling

Given our in vivo findings that LPS-induced neuroinflammation in the SN is accompanied by a marked reduction in microglial *O*-GlcNAcylation, we sought to investigate whether restoring *O*-GlcNAc levels directly in microglia could modulate inflammatory responses and inflammasome activation. Using primary microglial cultures, we observed that LPS stimulation significantly increased the expression of inflammation-related proteins, including iNOS and COX-2, as well as key inflammasome components such as NLRP3 and cleaved caspase-1 (Fig. S4A–C). LPS also induced a pro-inflammatory cytokine profile, characterized by elevated TNF-α, IL-1β, and IFN-γ, alongside a reduction in anti-inflammatory cytokines including IL-4, IL-10, and TGF-β (Fig. S4D). Treatment with GlcN, which enhances *O*-GlcNAcylation, effectively attenuated the LPS-induced upregulation of inflammatory and inflammasome markers and reversed the imbalance in cytokine expression (Fig. S4A–D). In parallel, LPS stimulation increased intracellular ROS levels and NOX2 protein expression in primary microglia, both of which were markedly attenuated by enhancement of *O*-GlcNAcylation (Fig. S4E, S4F). Similarly, Thiamet G treatment suppressed LPS-induced inflammatory signaling and inflammasome activation (Fig. S5A–D) and also reduced LPS-induced ROS accumulation and NOX2 expression (Fig. S5E, S5F). Consistent with the transcriptional changes observed in vivo, LPS-induced alterations in inflammasome components as well as pro- and anti-inflammatory cytokine transcripts were similarly reversed by GlcN or Thiamet G in primary microglia (Fig. S4G, S4H, S5G, S5H). Collectively, these findings indicate that enhancement of microglial O-GlcNAcylation is associated with modulation of inflammatory signaling, oxidative stress, and inflammasome-related responses under inflammatory conditions.

### Microglia-derived factors mediate neuronal cell loss

Because in vivo analyses do not distinguish whether neuronal TH loss is mediated by microglial factors, we employed a conditioned media approach to assess the effects of microglial secreted factors on neuronal injury. Primary microglia were treated with LPS, Thiamet G, or LPS plus Thiamet G, and the resulting conditioned media were applied to SH-SY5Y neuronal cultures. Conditioned media from LPS-stimulated microglia significantly reduced TH protein levels and neuronal viability in SH-SY5Y cells (Fig. S6A, S6B). In contrast, conditioned media from microglia co-treated with LPS and Thiamet G markedly attenuated these neurotoxic effects (Fig. S6A, S6B), indicating that modulation of microglial *O*-GlcNAcylation alters the neurotoxic properties of microglia-derived soluble factors.

### GlcN and Thiamet G Restore O-GlcNAcylation of NF-κB subunits and inhibit their nuclear accumulation

**Fig. 5:**
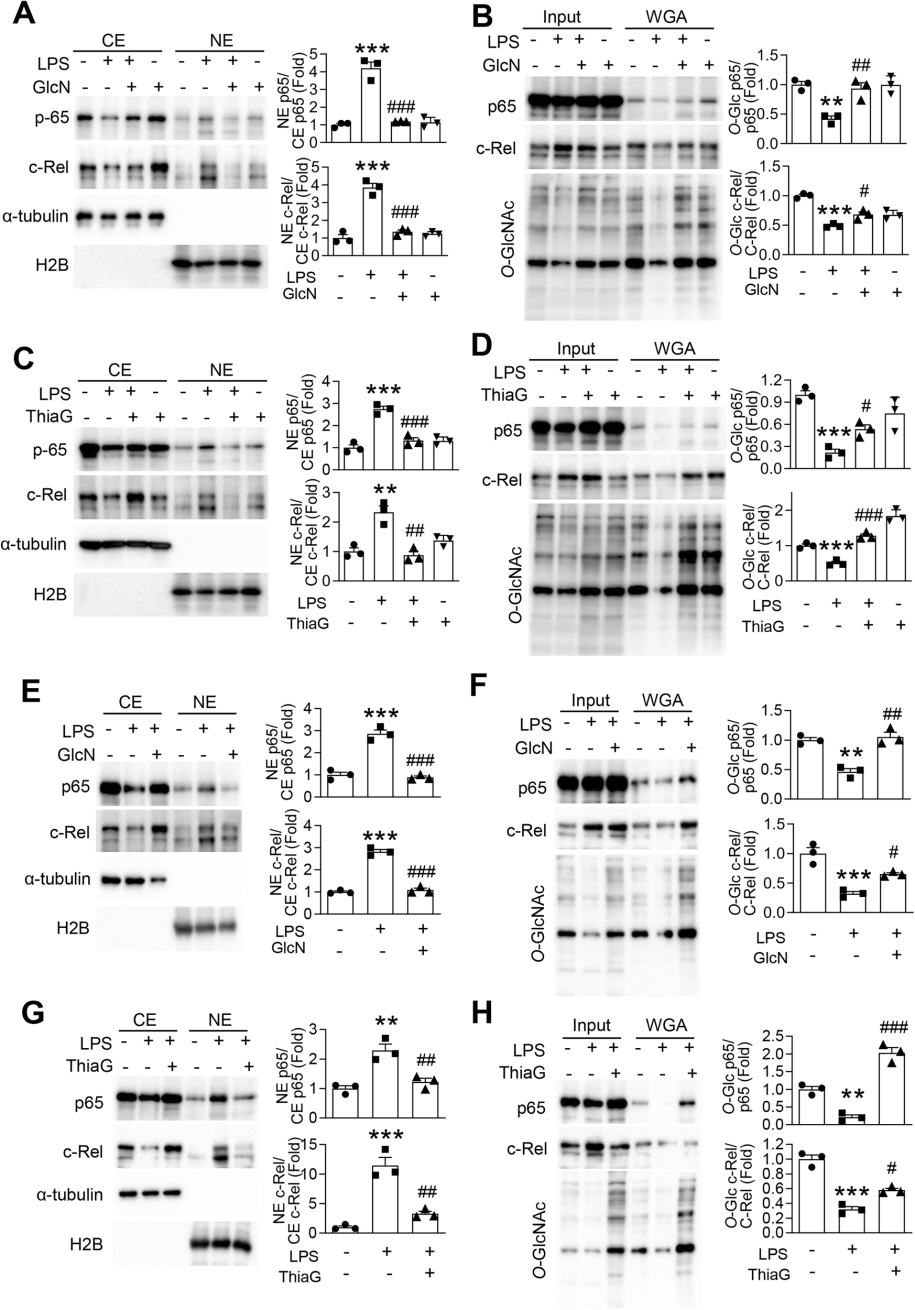
GlcN and Thiamet G modulates nuclear translocation of NF-κB subunits p65 and c-Rel in microglia. LPS (5 μg) was injected into the SN region of mouse brains. GlcN (200 mg/kg) or Thiamet G (ThiaG; 20 mg/kg) was administered intraperitoneally three times per week for 4 weeks (**A**–**D**). **A**, **C** Cytoplasmic (CE) and nuclear (NE) protein fractions were isolated from SN tissue. Western blot analysis was performed using antibodies against NF-κB subunits p65 and c-Rel. Nuclear levels of p65 and c-Rel were normalized to their corresponding cytoplasmic levels. H2B and α-tubulin were used as nuclear and cytoplasmic markers, respectively (*n* = 3/group). Wheat germ agglutinin (WGA) pull-down assays were performed using total SN protein lysates to assess *O*-GlcNAcylation of p65 and c-Rel. *O*-GlcNAc-modified forms were detected by western blot and normalized to total p65 or c-Rel levels (*n* = 3/group). **E**–**H** Primary microglial cells were treated with LPS (400 ng/mL) for 24 h in the presence or absence of GlcN (5 mM) or Thiamet G (ThiaG; 1 μM). **E**, **G** Nuclear and cytoplasmic fractions were analyzed for p65 and c-Rel distribution by western blot, with quantification normalized as described above (*n* = 3/group). **F**, **H** WGA pull-down assays were conducted on total cell lysates to assess *O*-GlcNAcylation of p65 and c-Rel (*n* = 3/group). Data are presented as mean SEM; ^*^p < 0.05, ^**^p < 0.01, ^***^p < 0.001 versus control, ^#^*p* < 0.05, ^##^*p* < 0.01, ^###^*p* < 0.001 versus LPS. Statistical analysis was performed using one-way ANOVA with Tukey’s post hoc multiple comparison test.

To elucidate the mechanism by which *O*-GlcNAcylation regulates microglial inflammatory responses, we investigated the nuclear translocation of NF-κB subunits p65 and c-Rel in the LPS-induced PD mouse model. Cytoplasmic and nuclear fractions were prepared from brain tissues, and the localization of p65 and c-Rel was assessed by western blot analysis. As expected, LPS administration led to a marked increase in the nuclear accumulation of both p65 and c-Rel in the brain compared to control animals, indicating activation of the NF-κB signaling pathway (Fig. 5A, C). In contrast, treatment with either GlcN or Thiamet G significantly attenuated the LPS-induced nuclear accumulation of p65 and c-Rel in the brain. Next, we assessed whether p65 and c-Rel undergo direct *O*-GlcNAc modification and whether this modification is affected by inflammatory stimuli. Precipitation with wheat germ agglutinin followed by western blotting revealed that *O*-GlcNAcylation levels of both p65 and c-Rel were significantly reduced in response to LPS stimulation (Fig. 5B, D). However, co-treatment with GlcN or Thiamet G restored the *O*-GlcNAcylation of these proteins. To confirm these findings in a cellular context, we performed similar experiments using primary microglial cultures. Consistent with in vivo results, LPS treatment induced robust nuclear translocation of p65 and c-Rel, which was significantly attenuated by GlcN or Thiamet G treatment (Fig. 5E, G). Moreover, LPS stimulation reduced the *O*-GlcNAcylation levels of p65 and c-Rel in microglial cells, which were restored by treatment with GlcN or Thiamet G (Fig. 5F, H). To further assess *O*-GlcNAcylation of individual NF-κB subunits, co-immunoprecipitation assays were performed in primary microglial cultures. Immunoprecipitation of p65 or c-Rel followed by immunoblotting with an *O*-GlcNAc antibody revealed a significant decrease in *O*-GlcNAcylation of both subunits in response to LPS, which was robustly reversed by GlcN or Thiamet G treatment (Fig. S7A–D). *O*-GlcNAc signals were normalized to the corresponding immunoprecipitated p65 or c-Rel levels. Collectively, these findings suggest that p65 and c-Rel are *O*-GlcNAc–modified in microglia and that modulation of *O*-GlcNAcylation correlates with changes in their nuclear translocation.

### Enhancement of O-GlcNAcylation modulates LPS-induced microglial activation

**Fig. 6:**
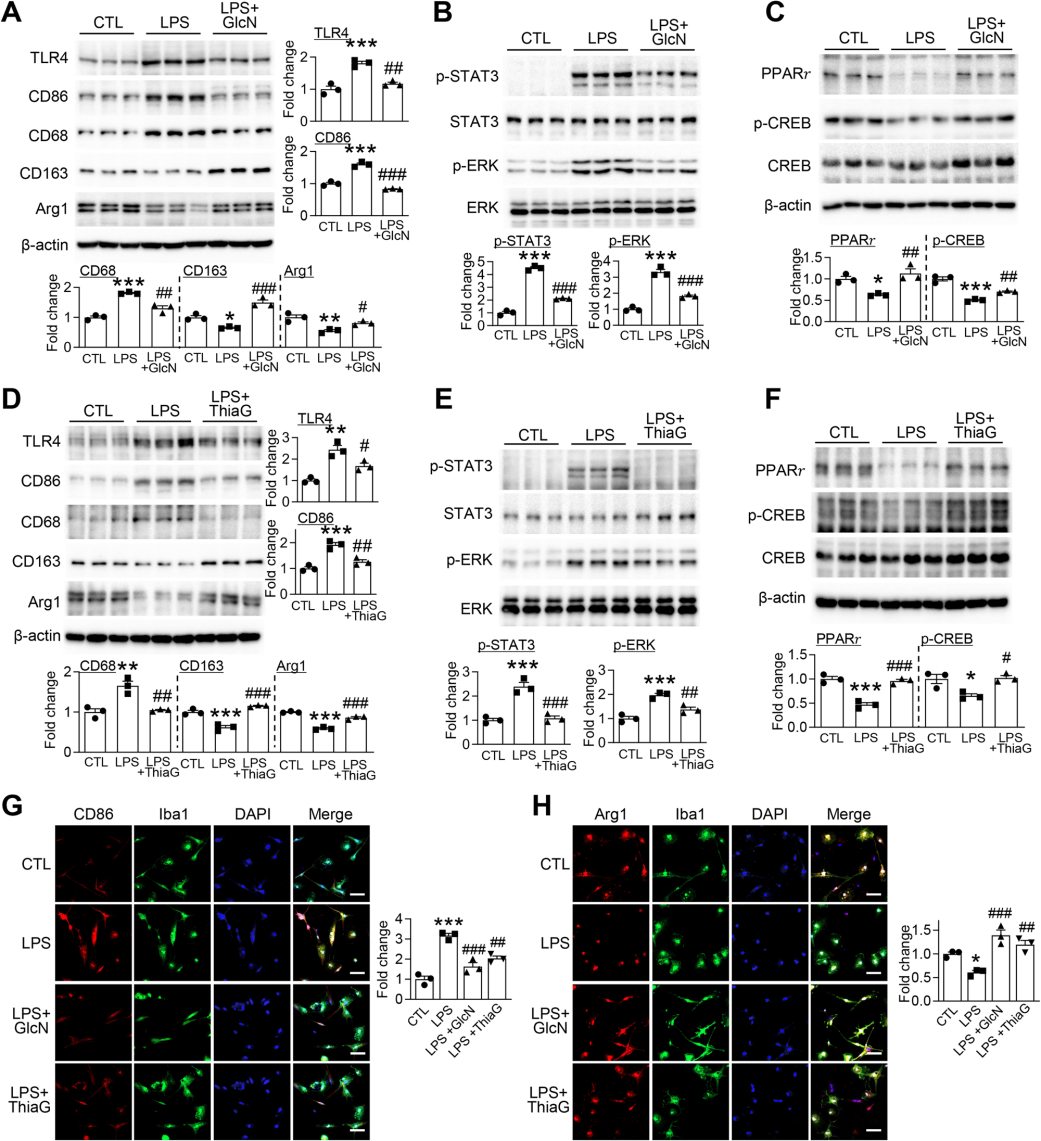
GlcN and Thiamet G enhance M2 polarization and suppress M1 signaling in microglia. Primary microglial cells were stimulated with LPS (400 ng/mL) for 24 h in the presence or absence of GlcN (5 mM) or Thiamet G (ThiaG; 1 μM) to assess phenotypic polarization and relevant signaling mechanisms. **A**, **D** Western blot analysis was performed to assess the expression of microglial polarization markers, including TLR4, CD86, CD68 (M1 markers), and CD163, Arg1 (M2 markers). β-actin was used as a loading control. Protein levels were quantified and normalized to β-actin (*n* = 3/group). **B**, **E** Expression of phosphorylated STAT3 (p-STAT3) and phosphorylated ERK (p-ERK) was analyzed along with their respective total protein levels. p-STAT3 and p-ERK expression was normalized to total STAT3 and ERK, respectively (*n* = 3/group). **C**, **F** Expression of PPARγ and phosphorylated CREB (p-CREB) was evaluated by western blot. p-CREB levels were normalized to total CREB, and PPARγ levels were normalized to β-actin (*n* = 3/group). **G** Representative immunofluorescence images of CD86 (red), Iba1 (green), and DAPI (blue) in primary microglial cells. Scale bar: 50 μm (*n* = 3/group). **H** Representative immunofluorescence images of Arg1 (red), Iba1 (green), and DAPI (blue) in primary microglial cells. Scale bar: 50 μm (*n* = 3/group). Data are presented as mean SEM; ^*^p < 0.05, ^**^p < 0.01, ^***^p < 0.001 versus control, ^#^*p* < 0.05, ^##^*p* < 0.01, ^###^*p* < 0.001 versus LPS. Statistical analysis was performed using one-way ANOVA with Tukey’s post hoc multiple comparison test.

Microglial activation is tightly regulated by NF-κB signaling and exhibits a spectrum of functional states in response to inflammatory stimuli^34,35^. Western blot analysis revealed that LPS treatment markedly increased the expression of pro-inflammatory markers, including TLR4, CD86, and CD68, while concurrently reducing the expression of markers associated with anti-inflammatory and neuroprotective functions, such as Arg1 and CD163 (Fig. 6A, D). At the signaling level, LPS stimulation enhanced the phosphorylation of STAT3 and ERK, indicative of activated inflammatory signaling (Fig. 6B, E), whereas regulatory molecules involved in anti-inflammatory and neuroprotective responses, including PPARγ and phosphorylated CREB, were diminished (Fig. 6C, F). Importantly, treatment with GlcN or Thiamet G significantly mitigated the LPS-induced pro-inflammatory activation. Both compounds suppressed the upregulation of inflammatory markers while restoring the expression of anti-inflammatory and neuroprotective molecules (Fig. 6A, D). These phenotypic changes were paralleled by molecular signaling alterations: GlcN and Thiamet G reduced phosphorylation of STAT3 and ERK (Fig. 6B, E), and enhanced PPARγ and p-CREB levels (Fig. 6C, F), indicating a shift toward a more balanced and neuroprotective activation state. Immunofluorescence staining corroborated these findings: LPS-induced increases in CD86 were attenuated, whereas the LPS-suppressed expression of Arg1 was restored following treatment (Fig. 6G, H). These results demonstrate that enhancing *O*-GlcNAcylation, either through GlcN supplementation or Thiamet G treatment, dampens pro-inflammatory microglial activation while promoting functional states associated with anti-inflammatory and neuroprotective responses.

### GlcN and Thiamet G modulate microglial activation and signaling pathways in the LPS-induced PD mouse brain

**Fig. 7:**
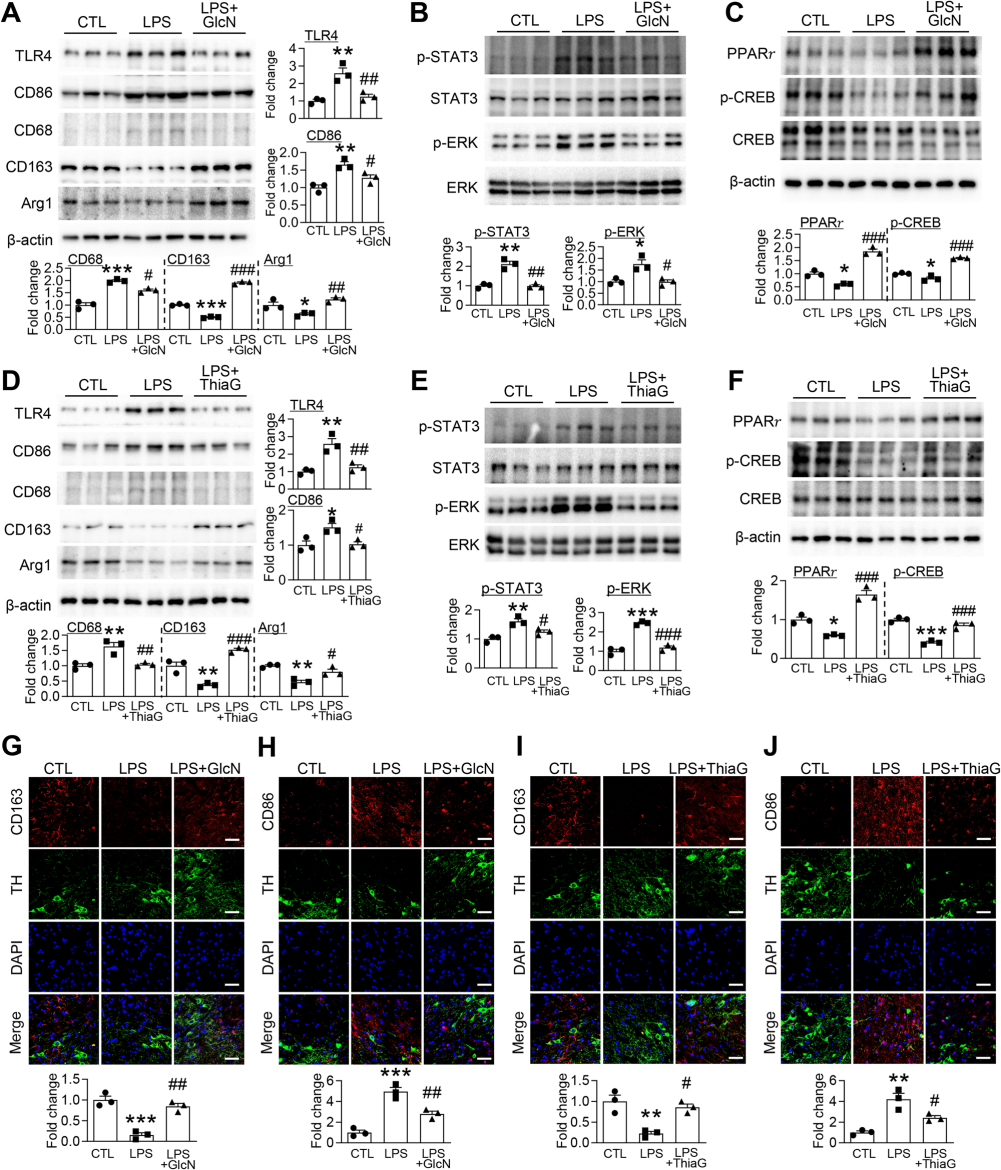
GlcN and Thiamet G promote anti-inflammatory microglial polarization in the SN of LPS-induced PD model mice. LPS (5 μg) was stereotaxically injected into the SN of mouse brains to induce neuroinflammation. GlcN (200 mg/kg) and Thiamet G (ThiaG; 20 mg/kg) were administered intraperitoneally three times per week for four weeks. **A**, **D** Western blot analyses of SN tissue lysates were performed to evaluate microglial polarization markers, including TLR4, CD86, CD68 (M1 markers), and CD163, Arg1 (M2 markers). β-actin was used as a loading control. Protein expression levels were quantified and normalized to β-actin (*n* = 3/group). **B**, **E** Phosphorylation levels of STAT3 and ERK were assessed using phospho-specific and total antibodies. The ratios of p-STAT3/STAT3 and *p*-ERK/ERK were calculated and normalized accordingly (*n* = 3/group). **C**, **F** PPARγ and p-CREB/CREB levels were evaluated by immunoblotting. Densitometric analyses were performed with normalization to β-actin or total CREB (*n* = 3/group). **G**, **I** Representative immunofluorescence images of CD163 (red), TH (green), and DAPI (blue) in the SN region to visualize anti-inflammatory (M2) microglial phenotypes. Scale bar: 50 μm (*n* = 3/group). **H**, **J** Representative immunofluorescence images of CD86 (red), TH (green), and DAPI (blue) in the SN region to visualize pro-inflammatory (M1) microglial phenotypes. Scale bar: 50 μm (*n* = 3/group). Data are presented as mean SEM; ^*^p < 0.05, ^**^p < 0.01, ^***^p < 0.001 versus control, ^#^*p* < 0.05, ^##^*p* < 0.01, ^###^*p* < 0.001 versus LPS. Statistical analysis was performed using one-way ANOVA with Tukey’s post hoc multiple comparison test.

To assess the effect of *O*-GlcNAcylation on microglial activation in vivo, we examined the expression of inflammatory and anti-inflammatory markers, as well as related signaling molecules, in the SN of LPS-induced PD model mice. Western blot analysis revealed that LPS injection markedly increased the expression of pro-inflammatory markers, including TLR4, CD86, and CD68, while simultaneously reducing the levels of molecules associated with anti-inflammatory and neuroprotective functions, such as Arg1 and CD163 (Fig. 7A, D). Administration of GlcN or Thiamet G effectively reversed these changes, significantly suppressing the upregulation of inflammatory markers and restoring the expression of protective molecules in the SN region of LPS-injected mice (Fig. 7A, D). Immunofluorescence staining further confirmed these observations. Following LPS treatment, microglia within the SN exhibited elevated immunoreactivity for inflammatory markers alongside reduced expression of anti-inflammatory markers (Fig. 7G–J). In contrast, GlcN and Thiamet G treatment attenuated inflammatory marker expression while enhancing the expression of anti-inflammatory and neuroprotective molecules (Fig. 7G–J). At the signaling level, LPS significantly increased phosphorylation of STAT3 and ERK—key mediators of pro-inflammatory activation—while suppressing PPARγ and phosphorylated CREB, which are associated with anti-inflammatory and neuroprotective responses (Fig. 7B, C, E, F). Notably, GlcN and Thiamet G treatments mitigated LPS-induced phosphorylation of STAT3 and ERK and restored PPARγ and p-CREB levels (Fig. 7B, C, E, F). Taken together, these results demonstrate that enhancing *O*-GlcNAcylation in the LPS-induced PD mouse model dampens pro-inflammatory microglial activation and signaling while promoting protective and anti-inflammatory microglial states in the SN.

### Modulation of O-GlcNAcylation levels regulates microglial activation states under basal conditions

**Fig. 8:**
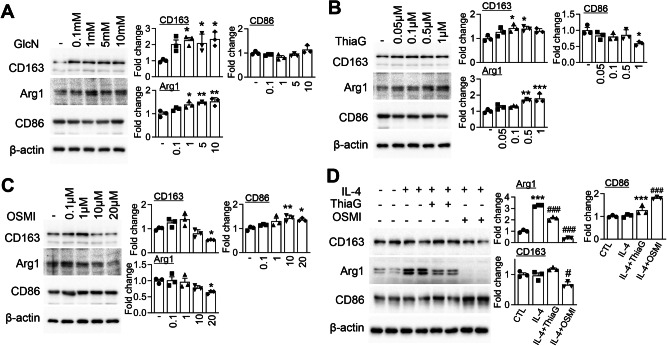
Modulation of *O*-GlcNAcylation levels regulates microglial differentiation toward M1 or M2 phenotypes. Primary murine microglial cells were treated with various concentrations of *O*-GlcNAc modulators to assess their effects on microglial polarization phenotypes. **A** Cells were treated with GlcN at concentrations of 0.1, 1, 5, and 10 mM for 24 h. Whole-cell lysates were analyzed by western blot using antibodies against CD86 (M1 marker), CD163, and Arg1 (M2 markers), and β-actin as a loading control. Densitometric quantification was performed and normalized to β-actin (*n* = 3/group). **B** Cells were treated with Thiamet G (ThiaG) at concentrations of 0.05, 0.1, 0.5, and 1 μM for 24 h. Western blot analyses and quantification were conducted as described in (**A**) (*n* = 3/group). **C** Cells were treated with OSMI-1 at concentrations of 0.1, 1, 10, and 20 μM for 24 h. Expression of CD86, CD163, and Arg1 was assessed via western blot and quantified relative to β-actin (*n* = 3/group). **D** To evaluate the effects of *O*-GlcNAc modulation compared to cytokine-induced polarization, cells were treated with IL-4 (20 ng/mL), Thiamet G (1 μM), or OSMI-1 (20 μM) for 24 h. Western blotting was performed for CD86, CD163, and Arg1 (*n* = 3/group). Data are presented as mean SEM; ^*^p < 0.05, ^**^p < 0.01, ^***^p < 0.001 versus control, ^#^*p* < 0.05, ^###^*p* < 0.001 versus IL-4. Statistical analysis was performed using one-way ANOVA with Tukey’s post hoc multiple comparison test.

To determine whether modulation of *O*-GlcNAcylation influences microglial functional states under non-inflammatory conditions, we manipulated *O*-GlcNAc cycling in primary microglial cultures using GlcN, Thiamet G, and OSMI-1 (an OGT inhibitor), and assessed their effects on microglial activation. Treatment with GlcN led to a dose-dependent increase in markers associated with anti-inflammatory (e.g., CD163 and Arg1) and neuroprotective functions, while pro-inflammatory marker expression remained largely unchanged (Fig. 8A). Thiamet G treatment similarly enhanced anti-inflammatory and protective marker expression and concurrently reduced pro-inflammatory markers (Fig. 8B). In contrast, OSMI-1 treatment produced the opposite effect, causing a dose-dependent decrease in anti-inflammatory markers and an increase in inflammatory markers, indicating that suppression of *O*-GlcNAcylation favors a pro-inflammatory microglial state (Fig. 8C). Western blot analysis confirmed that GlcN and Thiamet G elevated *O*-GlcNAcylation levels, whereas OSMI-1 reduced them in a dose-dependent manner (Fig. S8A–C), confirming the expected biochemical effects of these treatments. To further investigate the relationship between *O*-GlcNAcylation and microglial states, we examined the effects of altered *O*-GlcNAc levels on IL-4–induced anti-inflammatory activation. As expected, IL-4 stimulation significantly increased the expression of anti-inflammatory and neuroprotective markers (Fig. 8D). However, co-treatment with Thiamet G modestly attenuated this effect, suggesting that excessive *O*-GlcNAcylation may limit optimal protective activation (Fig. 8D). Notably, CD163 and CD86 levels remained unchanged under this condition. In contrast, reduction of *O*-GlcNAcylation by OSMI-1 abolished IL-4–induced expression of anti-inflammatory markers and enhanced pro-inflammatory marker expression, further supporting the role of *O*-GlcNAcylation in maintaining microglial functional balance (Fig. 8D). IL-4 stimulation alone caused a slight, non-significant increase in *O*-GlcNAcylation compared to controls (Fig. S8D). Collectively, these findings suggest that O-GlcNAcylation is associated with changes in microglial activation states under basal and cytokine-stimulated conditions.

## Discussion

PD is characterized not only by dopaminergic neuronal loss and α-synuclein aggregation but also by a sustained neuroinflammatory environment that contributes to disease progression^1,36,37^. Emerging evidence suggests that neuroinflammation in PD is tightly linked to metabolic dysregulation within the central nervous system, including altered glucose utilization, mitochondrial dysfunction, and aberrant nutrient-sensing pathways^38,39^. Among the key molecular mediators at the interface of metabolism and immune regulation is *O*-GlcNAcylation, governed by cellular nutrient status. *O*-GlcNAcylation has been shown to modulate inflammatory signaling both in the periphery and the central nervous system, acting as a homeostatic brake on excessive immune activation^26,28,29,31–33^.

In this study, we observed a significant reduction in protein *O*-GlcNAcylation in the SN of PD patients, with the most prominent decrease occurring in microglial cells. This reduction was associated with enhanced microglial activation, characterized by upregulation of inflammasome components and increased expression of pro-inflammatory cytokines. Although elevated inflammatory signaling in PD has been well documented previously^40–42^, our human data integrate established inflammatory features of PD into the context of altered microglial *O*-GlcNAc homeostasis, offering an additional regulatory layer underlying neuroinflammatory pathology. To extend these clinical observations and explore potential mechanistic links, we employed both an LPS-induced PD mouse model and primary microglial cultures. In these models, inflammatory stimulation consistently promoted a transition toward a neurotoxic microglial activation state, marked by elevated innate immune signaling, increased NLRP3 inflammasome activity, and loss of homeostatic microglial functions. Rather than reflecting a distinct activation subtype, this profile represents a continuum of pro-inflammatory microglial states that drive neurodegenerative processes. Importantly, these alterations coincided with a pronounced decline in protein *O*-GlcNAcylation, suggesting that disrupted *O*-GlcNAc signaling contributes to the persistence of microglial-mediated inflammation. These observations raise the question of whether inflammation-associated microglial states contribute to altered neuronal vulnerability. In line with our in vivo findings, conditioned media from LPS-stimulated microglia reduced TH expression and neuronal viability in SH-SY5Y cells, whereas enhancement of microglial *O*-GlcNAcylation with Thiamet G significantly attenuated these effects. Together, these data support a role for microglia-derived inflammatory factors in modulating dopaminergic neuronal integrity.

Previous studies using α-synuclein PFF or toxin-based Parkinson’s disease models have shown that pharmacological modulation of *O*-GlcNAcylation influences microglial activation and dopaminergic outcomes in proteinopathy- or toxin-driven contexts^43,44^. In such models, changes in tyrosine hydroxylase levels likely reflect downstream consequences of α-synuclein aggregation or direct neurotoxic injury. In contrast, the present study adopts an inflammation-driven paradigm in which dopaminergic vulnerability arises primarily from sustained microglial activation within the SN. Across experimental models and human PD tissue, we consistently observed a selective reduction of endogenous *O*-GlcNAcylation in microglia under inflammatory stress. Restoration of microglial *O*-GlcNAcylation was accompanied by shifts in inflammatory tone and microglial phenotype, identifying dysregulated microglial *O*-GlcNAc signaling as a distinct link between neuroinflammation and dopaminergic vulnerability.

Mechanistically, our data support a model in which enhanced *O*-GlcNAc signaling constrains inflammatory activation by modulating NF-κB–dependent transcription, potentially involving regulation of p65 and c-Rel and subsequent suppression of NLRP3 inflammasome components and pro–IL-1β expression. This interpretation is consistent with prior reports demonstrating that elevated *O*-GlcNAcylation attenuates NF-κB–mediated inflammatory responses in both neuroinflammatory and systemic inflammatory settings^31,32^. However, whether *O*-GlcNAcylation directly modifies NF-κB subunits at specific residues or acts indirectly through regulation of nuclear trafficking, chromatin interactions, or transcriptional co-factors remains unresolved and will require future studies using mass spectrometry–based site mapping and targeted mutagenesis.

Importantly, our data indicate that regulation of *O*-GlcNAc signaling under inflammatory stress cannot be explained solely by changes in the expression levels of OGT or OGA. In the SN, global *O*-GlcNAcylation did not strictly parallel the abundance of these enzymes. Notably, LPS-induced inflammation led to a marked reduction in *O*-GlcNAcylation without detectable changes in OGT or OGA protein levels, suggesting that inflammation-associated loss of *O*-GlcNAcylation may occur independently of transcriptional or translational regulation of the core cycling enzymes. Moreover, direct assessment of OGA activity revealed no increase sufficient to account for the magnitude of *O*-GlcNAc reduction. Together, these findings suggest that inflammatory stress may contribute to altered *O*-GlcNAc homeostasis, at least in part, by limiting upstream metabolic input into the HBP. Inflammatory activation has been reported to promote immunometabolic reprogramming toward aerobic glycolysis, a shift that could reduce the availability of glucose- and glutamine-derived substrates required for UDP-GlcNAc synthesis^45–47^. Accordingly, a relative reduction in HBP flux may represent one potential mechanism underlying the impaired *O*-GlcNAcylation observed during neuroinflammation. Further supporting a dissociation between enzyme expression and functional *O*-GlcNAc output, pharmacological inhibition of OGA with Thiamet G increased global *O*-GlcNAcylation despite concomitant upregulation of OGA mRNA and protein levels. This may represent a compensatory transcriptional response to maintain *O*-GlcNAc homeostasis despite suppressed enzymatic activity. Together, these observations highlight a dissociation between enzyme expression and modification status and underscore the complexity of *O*-GlcNAc regulation in the inflamed brain. Future studies directly measuring UDP-GlcNAc levels and assessing GFAT, OGT, and OGA enzymatic activity will be essential to delineate the relative contributions of these regulatory layers under neuroinflammatory and PD-relevant conditions.

Microglial activation has traditionally been described along a spectrum of functional states, ranging from pro-inflammatory and neurotoxic to anti-inflammatory and neuroprotective. In this study, we identify *O*-GlcNAcylation as an important molecular regulator of microglial activation states. To assess inflammation-associated changes in microglial state, we analyzed CD86, CD68, CD163, and Arg1, representing markers associated with pro- and anti-inflammatory phenotypic states. These markers were used to capture state-dependent shifts under altered *O*-GlcNAc signaling, rather than to imply a strict binary classification. Under inflammatory stimulation, microglia exhibited a pronounced shift toward a pro-inflammatory profile, characterized by enhanced innate immune signaling and suppression of genes associated with tissue repair and resolution. Remarkably, pharmacological elevation of *O*-GlcNAcylation effectively reversed this inflammatory bias, attenuating pro-inflammatory signaling while restoring the expression of genes linked to homeostatic and neuroprotective functions. These effects were consistently observed in both primary microglial cultures and the SN of LPS-induced PD model mice, suggesting that *O*-GlcNAcylation mitigates neuroinflammation by rebalancing microglial activation dynamics. Furthermore, modulation of *O*-GlcNAc levels under basal, non-inflammatory conditions was sufficient to alter microglial activation profiles, highlighting its intrinsic role in maintaining immune homeostasis. Agents that increased *O*-GlcNAcylation, such as GlcN and Thiamet G, promoted the expression of anti-inflammatory and reparative markers, whereas inhibition of OGT by OSMI-1 elicited the opposite effect, enhancing pro-inflammatory signaling. Interestingly, while moderate enhancement of *O*-GlcNAcylation supported homeostatic and protective microglial programs, excessive elevation—such as that induced by Thiamet G co-treatment during IL-4 stimulation—appeared to dampen the induction of reparative genes, suggesting a potential threshold beyond which *O*-GlcNAcylation may disrupt optimal microglial function. Conversely, suppression of *O*-GlcNAcylation abolished IL-4–induced expression of anti-inflammatory markers, reinforcing the notion that a basal level of *O*-GlcNAcylation is required for maintaining microglial equilibrium. Collectively, these findings position *O*-GlcNAcylation as a dynamic modulator of microglial functional plasticity, capable of constraining excessive inflammatory activation while supporting reparative and homeostatic programs. Future studies should aim to delineate the upstream metabolic cues and specific *O*-GlcNAc-modified targets that mediate this regulatory axis linking cellular metabolism to immune reprogramming in the brain.

Several limitations of our study warrant discussion. First, the number of human SN samples was limited by the availability of well-characterized postmortem tissue; therefore, the human data should be interpreted as supportive rather than definitive, and validation in larger cohorts will be required. Second, our study primarily relied on LPS- and toxin-based models that emphasize inflammatory pathology and may not fully capture α-synuclein–driven mechanisms in PD. Given emerging evidence that *O*-GlcNAcylation regulates α-synuclein folding and aggregation, future studies using genetic or α-synuclein–based models, including PFF injection or viral overexpression systems, will be important to integrate inflammation- and proteinopathy-driven processes. Accordingly, our findings should be interpreted within the context of inflammation-focused PD models. Third, systemic administration of GlcN or Thiamet G affects multiple cell types, making it difficult to ascribe observed effects specifically to microglia. Future studies using microglia-selective genetic tools (e.g., Cre-loxP–mediated OGT/OGA knockout or cell-targeted viral vectors) are needed to confirm cell-intrinsic effects. Fourth, while our data supports a role for *O*-GlcNAcylation in regulating microglial activation states, it is important to recognize that microglial phenotypes exist along a complex and dynamic spectrum that cannot be fully captured by simplified classifications. Consequently, more comprehensive profiling approaches, such as single-cell transcriptomics, will be necessary to precisely define how *O*-GlcNAcylation influences microglial heterogeneity, functional specialization, and the balance between pro-inflammatory and protective states. Fifth, long-term modulation of *O*-GlcNAcylation carries potential risks, given its widespread regulatory roles in diverse pathways, including insulin signaling and autophagy. Finally, although we focused on NF-κB, STAT3, and MAPK pathways, *O*-GlcNAc likely influences other components, such as RNA-binding proteins or additional inflammasome regulators, which remain to be identified.

In conclusion, our findings indicate that altered *O*-GlcNAcylation is associated with microglial activation and neuroinflammatory signaling in PD-relevant models. Using human tissue, in vivo, and in vitro approaches, we show that disruption of *O*-GlcNAc cycling accompanies inflammatory microglial states. These results support a role for *O*-GlcNAc signaling in the metabolic regulation of microglial inflammation and provide a foundation for future studies addressing its mechanistic and pathological significance in PD.

## Methods

### Primary mouse microglia isolation and culture

Cortices were isolated from postnatal day 0–3 (P0–P3) C57BL/6 mouse pups, and meninges were removed. Tissues were mechanically dissociated using a hand-held homogenizer and passed through a 25-gauge needle. The cell suspension was seeded into poly-L-lysine-coated T175 flasks (SPL Life Sciences, Korea) and cultured in DMEM/F12 with 10% FBS and 1% penicillin–streptomycin at 37 °C in 5% CO₂. Medium was changed every 3 days. After 10–14 days, microglia were isolated by gentle shaking (20–30 s), and the supernatant was centrifuged at 400 × *g* for 10 min. The resulting cell pellet was resuspended in fresh medium and counted for further experiments.

### Human brain tissue samples

The research resources (human brain tissue) were provided by the Korea Brain Bank Network (KBBN) operated through the National Brain Bank Project funded by the Ministry of Science and ICT of South Korea. Human brain tissues used for research purposes were approved by the Institutional Review Board of the Institutional Bioethics Committees, Inha University Hospital (approval number: 2023-05-018-000). Normal control brain samples included tissue from two females and one male donors (median age 65.3 years) with no significant Lewy body pathology upon neuropathological examination. PD brain samples comprised tissue from three female donors (median age 78.3 years) diagnosed clinically and confirmed neuropathologically.

### Whole protein lysate preparation

Human brain tissues, Mouse brain tissues (SN and striatum region) and cultured cells were homogenized in ice-cold RIPA buffer (Tech & Innovation, Chuncheon, Korea) supplemented with protease, phosphatase and *O*-GlcNAcase inhibitors (1 mM PMSF, 1 mM DTT, 1 mg/mL aprotinin, 1 mg/mL leupeptin, 1 mM sodium orthovanadate, 1 mM sodium fluoride, 1 mM streptozotocin). Homogenates or lysates were incubated on ice for 30 minutes with occasional vortexing and then centrifuged at 12,000 × *g* for 15 minutes at 4 °C to remove insoluble debris. The supernatant containing total protein (whole cell lysate) was collected, and protein concentration was determined by Bradford assay (Bio-Rad, Hercules, CA, USA).

### Cell culture

SH-SY5Y cells, an immortalized human neuronal cell line, were obtained from the Korean Cell Line Bank (Seoul, Korea). Cells were maintained in Dulbecco’s Modified Eagle’s Medium (DMEM; HyClone, UT, USA) supplemented with 10% fetal bovine serum (FBS; HyClone) and 100 U/mL penicillin-streptomycin (HyClone) under standard culture conditions. Cell line authentication was not performed. Cell lines were routinely screened for mycoplasma contamination to ensure experimental integrity and reproducibility. SH-SY5Y cells were differentiated with retinoic acid (RA, 10 µM, R2625, Sigma-Aldrich, St. Louis, MO, USA) for 5 days, with medium replaced every 2 days. Differentiated cells exhibited neuron-like morphology and were used for subsequent experiments.

### Cytoplasmic and nuclear fractionation

Freshly harvested microglia or mouse brain tissues were homogenized in ice-cold cytoplasmic extraction buffer (10 mM HEPES, 1.5 mM MgCl₂, 200 mM sucrose, 0.5% NP-40, 10 mM KCl) containing protease, phosphatase, and *O*-GlcNAcase inhibitors. After incubation on ice for 1 h, samples were centrifuged at 5000 rpm for 5 min at 4 °C to obtain cytoplasmic fractions (supernatant). The nuclear pellet was resuspended in nuclear extraction buffer (1 mM EDTA) and sonicated (3–5 cycles, 5 s each with 30 s intervals on ice). Nuclear extracts were clarified by centrifugation at 12,000 × *g* for 10 min at 4 °C. Protein concentrations were measured using the Bradford assay (Bio-Rad).

### Wheat Germ Agglutinin (WGA) pull-down assay

Protein lysates (500 μg) from mouse SN tissues or primary microglia were incubated with pre-cleared wheat germ agglutinin (WGA) agarose beads (Vector Laboratories, USA) at 4 °C for 24 h with gentle rotation. Beads were pre-washed with PBS and, after incubation, washed three times with ice-cold PBS to remove non-specific proteins. Bound glycoproteins were eluted by boiling in SDS-PAGE sample buffer for 5 min, then analyzed by SDS–PAGE and Western blotting using specific antibodies.

### OGA activity assay

Mouse brain tissues (SN) were lysed in lysis buffer A (150 mM NaCl, 50 mM Tris-pH 8.0, 0.5% NP-40) containing protease and phosphatase inhibitors. Samples (100 μg) were mixed with OGA activity buffer (50 mM cacodylate, pH 6.4, 50 mM D-GalNAc, 0.3% BSA, 1 mM FD-GlcNAc) and incubated at 37 °C for 3 h. The reaction was stopped by the addition 0.5 M Na_2_CO_3_. Absorbance was measured at an excitation wavelength of 485 nm and an emission wavelength of 535 nm.

### Western Blotting

Equal amounts of protein (30 μg) were separated on 10% or 15% SDS-PAGE gels and transferred to nitrocellulose membranes (Amersham). Membranes were blocked with 5% nonfat milk in PBST for 1 h at room temperature, then incubated overnight at 4 °C with primary antibodies (1:1000) against various targets, including *O*-GlcNAc (sc-59623, RL2, Santa Cruz, Dallas, TX, USA), OGT (sc-74546, Santa Cruz), OGA (14771-1-AP, Proteintech, Rosemont, IL, USA), GFAT2 (A15374, Abclonal, Woburn, MA, USA) tyrosine hydroxylase (T2928,TH, Sigma-Aldrich), β-actin (sc-28365, Santa Cruz), p-p65 (ab86229, Abcam, Cambridge, UK), p65 (sc-8008, Santa Cruz), p-IκB (sc-8404, Santa Cruz), IκB (sc-371, Santa Cruz), iNOS (610328, BD Bioscience, San Jose, CA, USA), COX2 (A3560, Abclonal), p-CREB (ab32096, Abcam), CREB (sc-377154, Santa Cruz), TLR4 (A0007, Abclonal), Arg1 (A4923, Abclonal), CD163 (A26411PM, Abclonal), CD86 (A16805, Abclonal), CD63 (A6554, Abclonal), c-Rel (sc-6955, Santa Cruz), p-STAT3 (9145, Cell Signaling Technology, Danvers, MA, USA), STAT3 (sc-8019, Santa Cruz), p-ERK (9101, Cell Signaling Technology), ERK (A16686, Abclonal), PPARγ (sc-7273, Santa Cruz), NLRP3 (sc-134306, Santa Cruz), ASC (sc-514414, Cell Signaling Technology), cleaved caspase 1 (89332, Cell Signaling Technology), and caspase 1 (24232, Cell Signaling Technology). Membranes were washed and incubated with horseradish peroxidase-conjugated secondary antibodies (Invitrogen) for 1 h at RT. Protein bands were visualized using an enhanced chemiluminescence detection system (Bio-Rad) and quantified by densitometry when needed.

### Immunohistochemistry and immunocytochemistry

Primary microglia cultured on poly-L-lysine-coated coverslips and 30 μm-thick brain sections were washed with PBST (0.05% Triton X-100 in PBS) and blocked with 0.5% BSA in PBST for 2 h at room temperature. Samples were incubated overnight at 4°C with primary antibodies in blocking buffer: anti-TH (T2928, Sigma-Aldrich), anti-*O*-GlcNAc (sc-59623, RL2, Santa Cruz), anti-GFAP (G3893, Sigma-Aldrich), anti-Iba1 (178846, Abcam), anti-CD163 (A26411PM, Abclonal), anti-CD86 (A16805, Abclonal), and anti-ASC (sc-514414, Santa Cruz) (all 1:100). After washing, Alexa Fluor-conjugated secondary antibodies (Invitrogen) were applied for 1 h at room temperature in the dark. Nuclei were counterstained with DAPI (Invitrogen).

For TH immunohistochemistry, sections were incubated with biotinylated secondary antibodies and visualized using DAB (Vector Laboratories). DAB-stained sections were dehydrated and imaged using bright-field microscopy (Olympus, Tokyo, Japan). Confocal images were acquired using a Zeiss LSM 980 confocal microscope (Zeiss, Oberkochen, Germany), and image analysis was performed using ImageJ (NIH) or ZEN software.

### Inflammatory cytokines measurement

Levels of inflammatory cytokines in mouse brain and cell lysates were quantified using commercially available ELISA kits (Arigo Biolaboratories, Hsinchu, Taiwan) following the manufacturer’s protocols. Briefly, brain and cell lysates were diluted in assay buffer to fall within the standard curve range. Samples and standards were added to cytokine antibody-coated plates and incubated overnight at 4 °C. After washing, enzyme-linked detection antibodies were applied. Following additional washes, substrate was added for 20 min, and the reaction was stopped. Absorbance was measured at 450 nm using a microplate reader (SpectraMax, Molecular Devices).

### Intracellular ROS and Hydrogen peroxide (H₂O₂) measurement

Cells were incubated with the ROS detection reagent (DCFDA) supplied in the ROS assay kit (BioMax, Korea) in serum-free medium at 37 °C for the manufacturer-recommended duration, protected from light. After washing to remove excess dye, cells were treated with the indicated conditions. Fluorescence was measured using a microplate reader (Ex/Em = 485/535 nm, SpectraMax, Molecular Devices). H₂O₂ levels were measured in SN tissue lysates using a fluorescence-based H₂O₂ assay kit according to the manufacturer’s instructions (Sigma-Aldrich). For each reaction, up to 50 µL of tissue lysate was added to a 96-well plate and adjusted to a final volume of 50 µL with assay buffer. Subsequently, 50 µL of the reaction master mix was added to each well containing samples, standards, or controls. Plates were gently mixed and incubated at room temperature for 15–30 minutes, protected from light. Fluorescence was measured using a microplate reader at an excitation wavelength of 540 nm and an emission wavelength of 590 nm. Data was normalized to protein concentration.

### Quantitative real-time PCR

Total RNA was isolated from tissues using TRIzol reagent (Ambion, Thermo Fisher Scientific) according to the manufacturer’s instructions. RNA concentration and purity were assessed by spectrophotometry, and 1 µg of total RNA was reverse-transcribed into cDNA using the Promega GoScript reverse transcription system (Promega Corp., Madison, USA). Quantitative PCR was performed using a SYBR Green-based master mix (BioFACT, Daejeon, Korea) on a real-time PCR system (Bio-Rad). Amplification conditions were as follows: initial denaturation at 95 °C for 2 min, followed by 40 cycles of 95 °C for 15 s and 60 °C for 60 s. Melt-curve analysis was conducted to confirm amplification specificity. Relative gene expression was calculated using the 2^−ΔΔCt method, with target gene Ct values normalized to an internal reference gene (GAPDH). Data are presented as fold change relative to the control group. Primer sequences were as follows: NLRP3 forward, 5′-CAGGCATCGGGAAAACCATC-3′; reverse, 5′-AGGATCTTGCACACTGGTGG-3′; ASC forward, 5′-TGACTGTGCTTAGAGACATGGG-3′; reverse, 5′-AAAGCATCCAGCCACTCCGT-3′; TNFα forward, 5′-AGGCACTCCCCCAAAGATG-3′; reverse, 5′-TGAGGGTCTGGGCCATAGAA-3′; IFN-γ forward, 5′-CCACGGCACAGTCATTGAAA -3′; reverse, 5′-TGCTGATGGCCTGATTGTCTT-3′; IL-1β forward, 5′-TGGTGTGTGACGTTCCCATT-3′; reverse, 5′-TGTCGTTGCTTGGTTCTCCT-3′; TGFβ forward, 5′-GCTGCGCTTGCAGAGATTAAA-3′; reverse, 5′-TCGAAAGCCCTGTATTCCGT-3′; IL-4 forward, 5′- -3′; reverse, 5′- -3′; IL-10 forward, 5′-CAGTACAGCCGGGAAGACAA-3′; reverse, 5′-AGGCTTGGCAACCCAAGTAA-3′; IL-4 forward, 5′-ACAGGAGAAGGGACGCCAT-3′; reverse, 5′-GAAGCCCTACAGACGAGCTCA -3′.

### Experimental animals and drug administration

All animal experiments were approved by the Institutional Animal Care and Use Committee of Inha University (Approval Number: 190920-655) and conducted in accordance with institutional guidelines. Male C57BL/6J mice (7 weeks old; DBL, Chungbuk, Korea) were housed under controlled conditions with a 12-hour light/dark cycle, ambient temperature (22 ± 2°C), and ad libitum access to food and water. Mice were acclimated for one week prior to experimentation. To induce neuroinflammation and dopaminergic neuron degeneration, lipopolysaccharide (LPS; 5 μg in 2 μL sterile saline; L4391, Sigma-Aldrich) was stereotaxically injected into the SN using the following coordinates relative to bregma: AP -3.1 mm, ML ± 1.5 mm, and DV -4.5 mm from the brain surface. Injections were performed with a 26-gauge stainless steel needle attached to a 10 μL Hamilton microsyringe at a slow rate. After injection, the needle was kept in place for 10 minutes before gradual withdrawal to prevent reflux. GlcN (200 mg/kg; G4875, Sigma-Aldrich) and Thiamet G (20 mg/kg; HY12588, MCE, Monmouth Junction, NJ, USA) were intraperitoneal injected three times weekly for four weeks, starting one day after LPS injection. Control animals received equivalent volumes of vehicle (sterile saline).

### Behavioral assessments

All apparatuses were cleaned with 70% ethanol between trials to eliminate olfactory cues. Tests were conducted during the light phase (09:00–17:00 h) under consistent lighting and noise conditions.

### Pole Test

To assess bradykinesia and coordination, mice were tested on a 50cm-high, 1cm-diameter vertical pole wrapped with gauze for grip. After five acclimation trials the day before, each mouse was placed head-up on the pole, and the time to turn and descend was recorded. Three trials were performed per mouse with 2-minute intervals. If a mouse failed to descend within 120 s, a maximum time of 120 s was recorded. The average of the three trials was used for analysis.

### Rotarod test

Motor coordination and balance were evaluated using an accelerating Rotarod (MED Associates). Mice were habituated at 5 rpm for 15 min one day before testing. During training (days 1–3), mice underwent two daily sessions at 20 rpm (max 10 min), with at least 30 min between sessions. On test day (day 4), the rod accelerated from 5 to 40 rpm over 300 s, then remained at 30 rpm for another 300 s. Each mouse performed three trials with 20 min intervals. Latency to fall was recorded, and the average was used for analysis.

### Statistical analyses

Data are shown as mean ± SEM from at least three independent experiments, with sample sizes in figure legends. Two-group comparisons used unpaired two-tailed Student’s t-tests. For three or more groups, one-way ANOVA followed by Tukey’s post-hoc test was applied. Analyses were done using GraphPad Prism 10.0 (GraphPad Software, CA, USA), and p < 0.05 was considered significant.

## Supplementary information

41531_2026_1319_MOESM1_ESM

41531_2026_1319_MOESM2_ESM

## Data Availability

The data and materials used in this research are available upon request from the corresponding author.
