# Supplementary material for: O-GlcNAcylation regulates microglial neuroinflammation in Parkinson’s disease
Source: NPJ Parkinsons Dis. 2026 Mar 28;12:121. doi: 10.1038/s41531-026-01319-6 (PMC13194708; doi:10.1038/s41531-026-01319-6)
Supplement: Supplementary file 1 — 41531_2026_1319_MOESM1_ESM [file 41531_2026_1319_MOESM1_ESM.pdf]

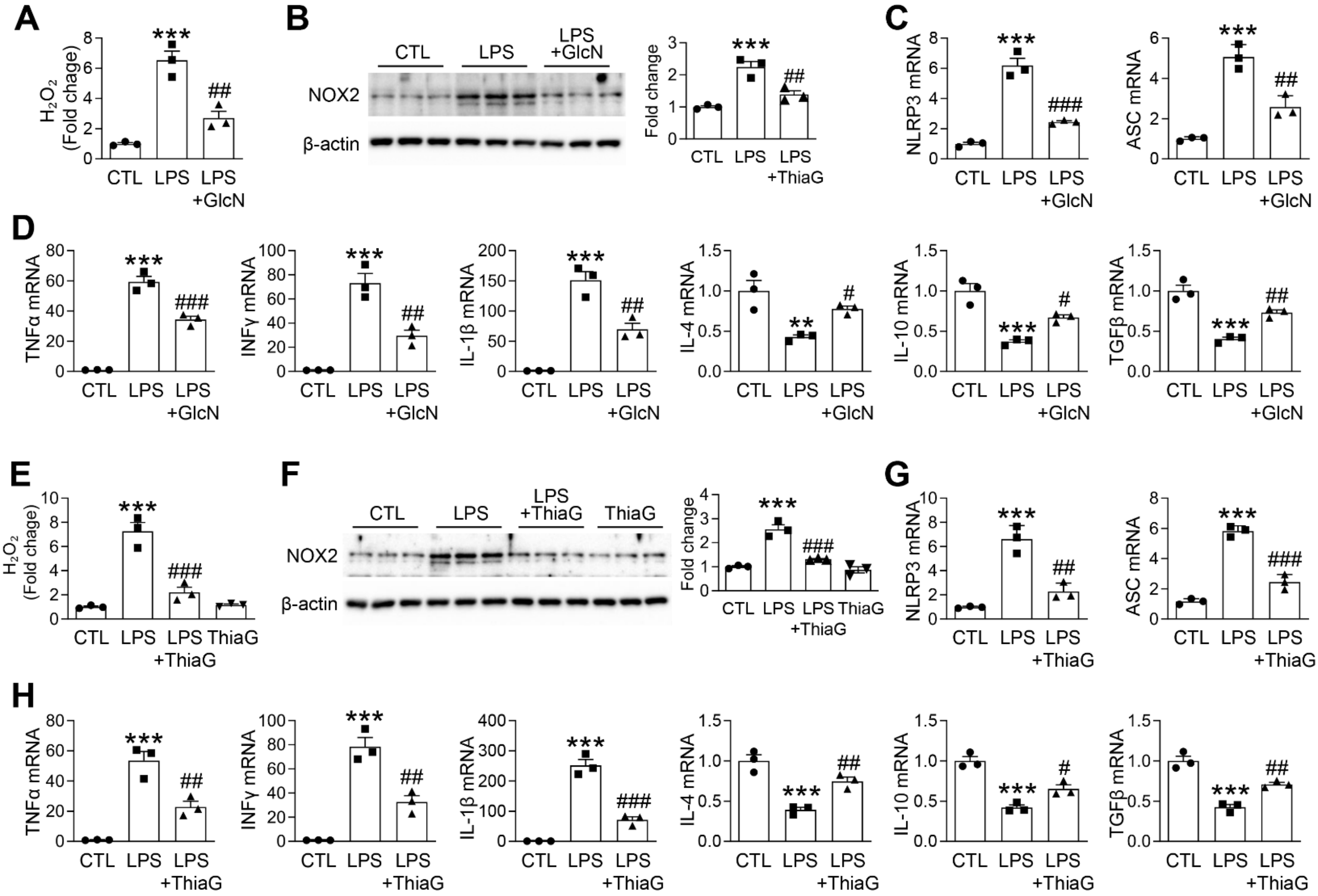

Supplementary Figure 1. Assessment of oxidative stress and inflammatory transcriptional responses in the SN

LPS (5 µg) was stereotaxically injected into the SN of mouse brains to induce neuroinflammation. GlcN (200 mg/kg) and Thiamet G (ThiaG; 20 mg/kg) were administered intraperitoneally three times per week for four weeks. (A, E) Quantification of hydrogen peroxide (H_2O_2) levels in SN tissue using a fluorescence-based H_2O_2 assay kit (n = 3 per group). (B, F) Representative immunoblot and quantitative analysis of NOX2 protein expression, with signal intensity normalized to β-actin (n = 3 per group). (C, D, G, H) Analysis of inflammatory gene transcript levels in SN tissue, including inflammasome-related genes and pro- and anti-inflammatory cytokines. Data are presented as fold change relative to control (n = 3 per group). Data are presented as mean SEM; $^{***}p<0.001$ versus control, $^{\#}p<0.05$, $^{\#\#}p<0.01$, $^{\#\#\#}p<0.001$ versus LPS. Statistical analysis was performed using one-way ANOVA with Tukey's post hoc multiple comparison test.

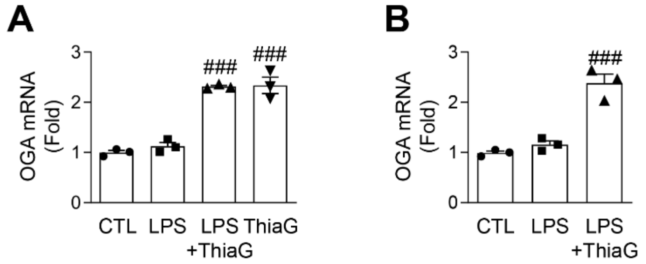

Supplementary Figure 2. Thiamet G increases *Oga* transcript levels *in vivo* and *in vitro*

(A) LPS (5 µg) was stereotaxically injected into the SN of mouse brains to induce neuroinflammation. Thiamet G (ThiaG; 20 mg/kg) were administered intraperitoneally three times per week for four weeks. Analysis of *Oga* gene transcript levels in the SN tissue. Data are presented as fold change relative to control (n = 3 per group). (B) Primary microglial cells treated with LPS (400ng/ml) with or without Thiamet G (1µM) for 24h. Analysis of *Oga* gene transcript levels in the microglial cells. Data are presented as fold change relative to control (n = 3 per group). Data are presented as mean SEM; ###$p<0.001$ versus LPS. Statistical analysis was performed using one-way ANOVA with Tukey's post hoc multiple comparison test.

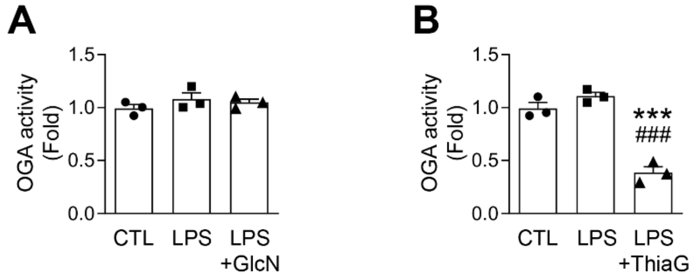

Supplementary Figure 3. Assessment of OGA enzymatic activity following LPS-induced inflammatory stress in the SN

LPS (5 µg) was stereotaxically injected into the substantia nigra (SN) of mouse brains to induce neuroinflammation. Glucosamine (GlcN; 200 mg/kg) and Thiamet G (ThiaG; 20 mg/kg) were administered intraperitoneally three times per week for four weeks. (A, B) OGA enzymatic activity in SN tissue was measured using a fluorescence-based OGA activity assay (n = 3 per group). Data are presented as fold change relative to control (n = 3 per group). Data are presented as mean SEM; $^{***}p<0.001$ versus control, $^{\#\#\#}p<0.001$ versus LPS. Statistical analysis was performed using one-way ANOVA with Tukey's post hoc multiple comparison test.

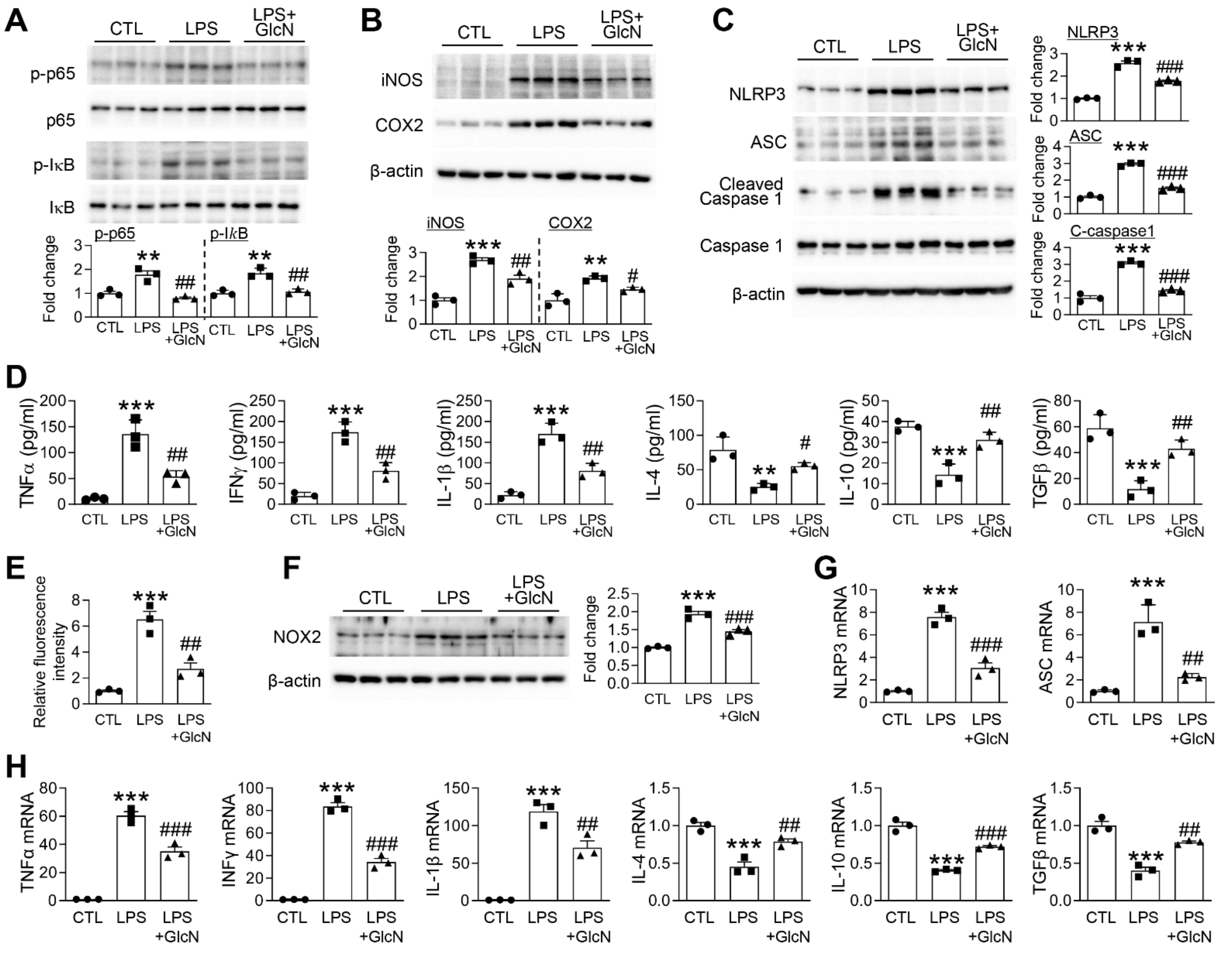

Supplementary Figure 4. GlcN regulates LPS-driven inflammation cascade and inflammasome responses in microglia

Primary microglial cells were stimulated with LPS (400 ng/mL) for 24 hours in the presence or absence of GlcN (5 mM). (A) Western blot analysis of NF-κB signaling components, including phosphorylated and total p65 and IκB, with phosphorylation levels normalized to total protein. (B) Expression of pro-inflammatory enzymes iNOS and COX-2 normalized to β-actin. (C) Assessment of inflammasome activation by measuring NLRP3, ASC, and cleaved caspase-1 relative to total caspase-1 and β-actin. (D) ELISA quantification of pro-inflammatory cytokines (TNF-α, IFN-γ, IL-1β) and anti-inflammatory cytokines (IL-4, IL-10, TGF-β) in culture supernatants. (E) Quantification of ROS using a fluorescence-based ROS assay kit (n = 3 per group). (F) Western blot analysis of NOX2 protein expression, with signal intensity normalized to β-actin (n = 3 per group). (G, H) Analysis of inflammatory gene transcript levels in microglial cells, including inflammasome-related genes and pro- and anti-inflammatory cytokines. Data are presented as fold change relative to control (n = 3 per group). Data are presented as mean SEM; $^{**}p<0.01$, $^{***}p<0.001$ versus control, $^{\#}p<0.05$, $^{\#\#}p<0.01$, $^{\#\#\#}p<0.001$ versus LPS. Statistical analysis was performed using one-way ANOVA with Tukey's post hoc multiple comparison test.

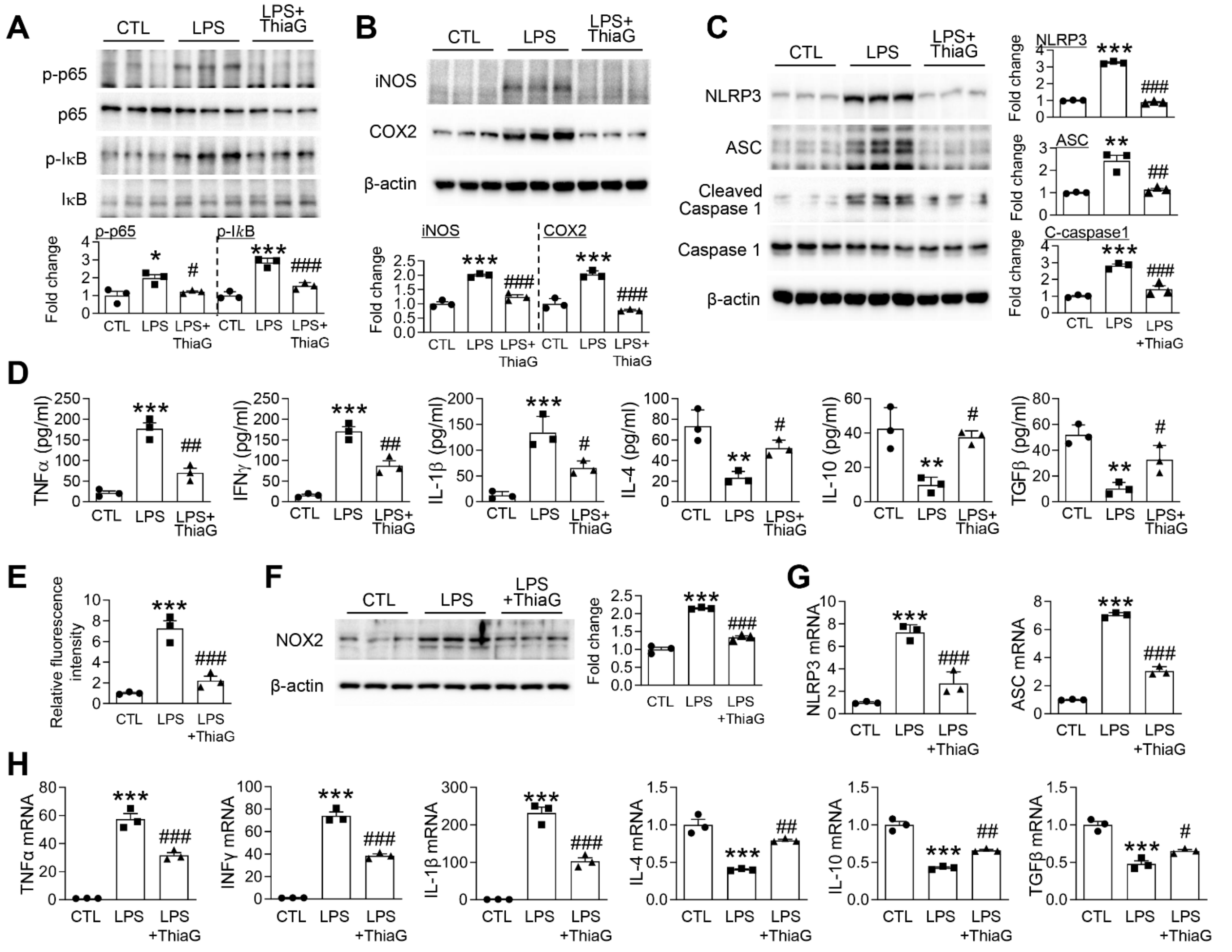

Supplementary Figure 5. Thiamet G regulates LPS-induced inflammatory and inflammasome responses in microglial cells

Primary microglial cells were stimulated with LPS (400 ng/mL) for 24 hours in the presence or absence of Thiamet G (ThiaG, 1 μM). (A) Western blot analysis of NF-κB signaling components, including phosphorylated and total p65 and IκB, with phosphorylation levels normalized to total protein. (B) Expression of pro-inflammatory enzymes iNOS and COX-2 normalized to β-actin. (C) Assessment of inflammasome activation by measuring NLRP3, ASC, and cleaved caspase-1 relative to total caspase-1 and β-actin. (D) ELISA quantification of pro-inflammatory cytokines (TNF-α, IFN-γ, IL-1β) and anti-inflammatory cytokines (IL-4, IL-10, TGF-β) in culture supernatants. (E) Quantification of ROS using a fluorescence-based ROS assay kit (n = 3 per group). (F) Western blot analysis of NOX2 protein expression, with signal intensity normalized to β-actin (n = 3 per group). (G, H) Analysis of inflammatory gene transcript levels in microglial cells, including inflammasome-related genes and pro- and anti-inflammatory cytokines. Data are presented as fold change relative to control (n = 3 per group).Data are presented as mean SEM; **p<0.01, ***p<0.001 versus control, $^{\#}$p<0.05, $^{\#\#}$p<0.01, $^{\#\#\#}$p<0.001 versus LPS. Statistical analysis was performed using one-way ANOVA with Tukey's post hoc multiple comparison test.

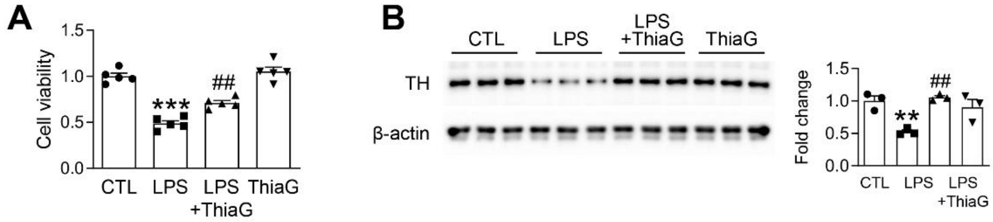

Supplementary Figure 6. Microglia-Derived Factors Mediate Neuronal cell Loss

Differentiated SH-SY5Y cells were treated with conditioned media collected from primary microglia exposed to LPS, Thiamet G, or LPS plus Thiamet G, as indicated. (A) Quantification of neuronal cell viability following conditioned media treatment (n = 5 per group). (B) Representative immunoblot and quantitative analysis of TH protein expression in differentiated SH-SY5Y cells, with signal intensity normalized to the indicated loading control (n = 3 per group). Data are presented as mean SEM; $^{***}p<0.001$ versus control, $^{\#\#}p<0.01$, $^{\#\#\#}p<0.001$ versus LPS. Statistical analysis was performed using one-way ANOVA with Tukey's post hoc multiple comparison test.

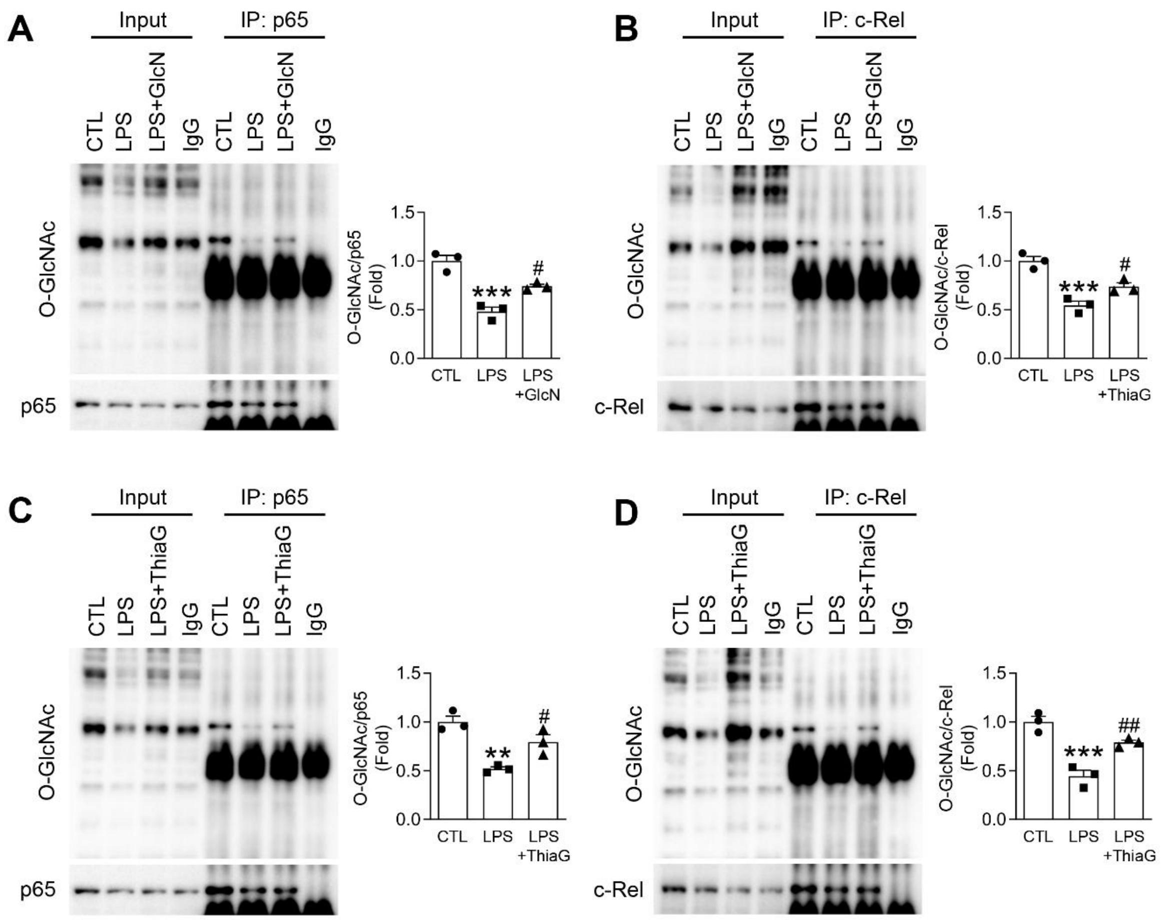

Supplementary Figure 7. Inflammatory stress alters *O*-GlcNAcylation of p65 and c-Rel in primary microglial cells

Primary microglial cells were treated with LPS (400 ng/mL) for 24 h in the presence or absence of GlcN (5 mM) or Thiamet G (ThiaG; 1 μM). (A, C) p65 was immunoprecipitated from whole-cell lysates, followed by immunoblotting with a *O*-GlcNAc antibody to assess *O*-GlcNAcylation of p65. *O*-GlcNAc signals were normalized to the amount of immunoprecipitated p65 (n = 3/group). (B, D) c-Rel was immunoprecipitated from the same experimental conditions, and *O*-GlcNAcylation of c-Rel was detected by immunoblotting with a *O*-GlcNAc antibody. *O*-GlcNAc signals were normalized to the amount of immunoprecipitated c-Rel (n = 3/group). Data are presented as mean SEM; $^{**}p<0.01$, $^{***}p<0.001$ versus control, $^{\#}p<0.05$, $^{\#\#}p<0.01$, versus LPS. Statistical analysis was performed using one-way ANOVA with Tukey's post hoc multiple comparison test.

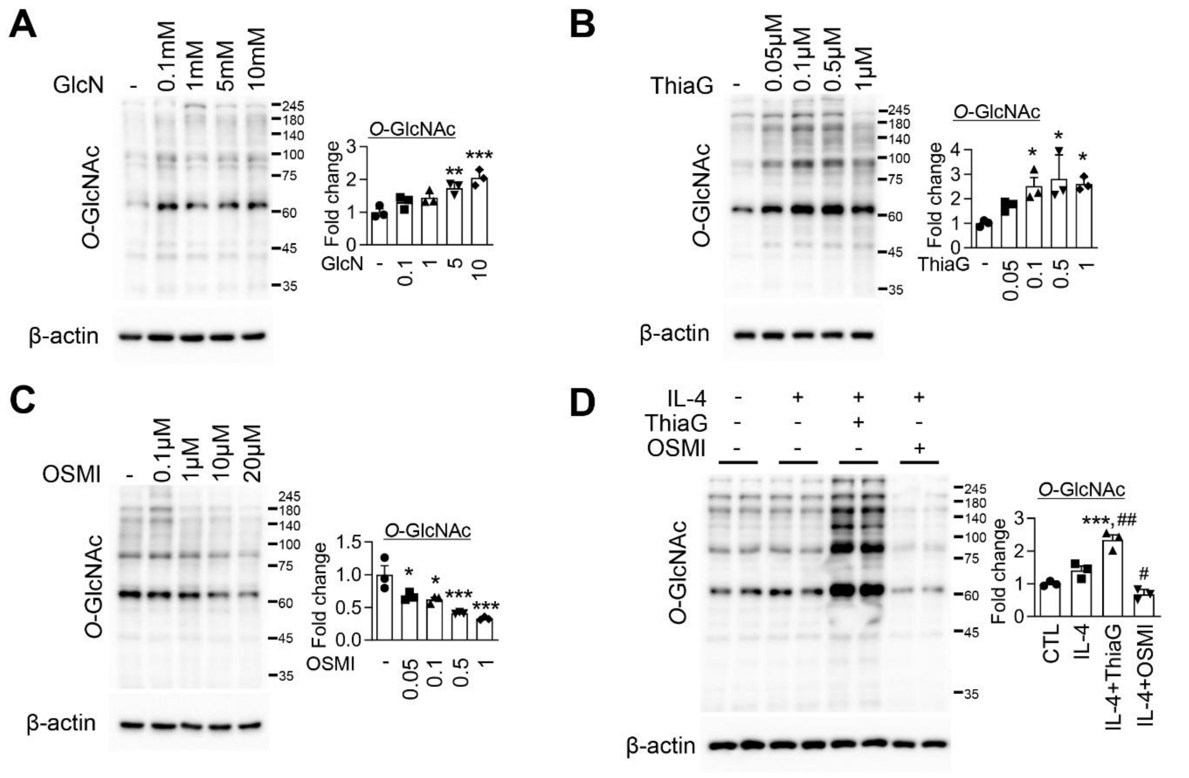

Supplementary Figure 8. Dose-dependent regulation of protein *O*-GlcNAcylation by GlcN, Thiamet G, and OSMI-1 in primary microglial cells

Primary murine microglial cells were treated with *O*-GlcNAc-modulating compounds to examine their effects on global *O*-GlcNAcylation levels. (A) Cells were exposed to GlcN at concentrations of 0.1, 1, 5, and 10 mM for 24 h. Whole-cell lysates were analyzed via western blot using anti-*O*-GlcNAc antibody. β-actin served as a loading control. Quantitative analysis was performed by normalizing *O*-GlcNAc signal to β-actin (n = 3/group). (B) Cells were treated with Thiamet G (ThiaG) at concentrations of 0.05, 0.1, 0.5, and 1 μM for 24 h. Western blotting and densitometric analysis were performed as in (E) (n = 3/group). (C) Cells were treated with OSMI-1 at concentrations of 0.1, 1, 10, and 20 μM for 24 h. *O*-GlcNAc expression was analyzed via western blot and quantified relative to β-actin (n = 3/group). (D) To compare the effects of *O*-GlcNAc modulation with cytokine-induced signaling, cells were exposed to IL-4 (20 ng/mL), Thiamet G (1 μM), or OSMI-1 (20 μM) for 24 h. Representative immunoblots showing total *O*-GlcNAcylation and β-actin levels are presented (n = 3/group). Data are presented as mean SEM; $^{*}p<0.05$, $^{**}p<0.01$, $^{***}p<0.001$ versus control, $^{\#}p<0.05$, $^{\#\#}p<0.01$, $^{\#\#\#}p<0.001$ versus IL-4. Statistical analysis was performed using one-way ANOVA with Tukey's post hoc multiple comparison test.
