# Supplementary material for: O-GlcNAcylation regulates microglial neuroinflammation in Parkinson’s disease
Source: NPJ Parkinsons Dis. 2026 Mar 28;12:121. doi: 10.1038/s41531-026-01319-6 (PMC13194708; doi:10.1038/s41531-026-01319-6)

Figure 1

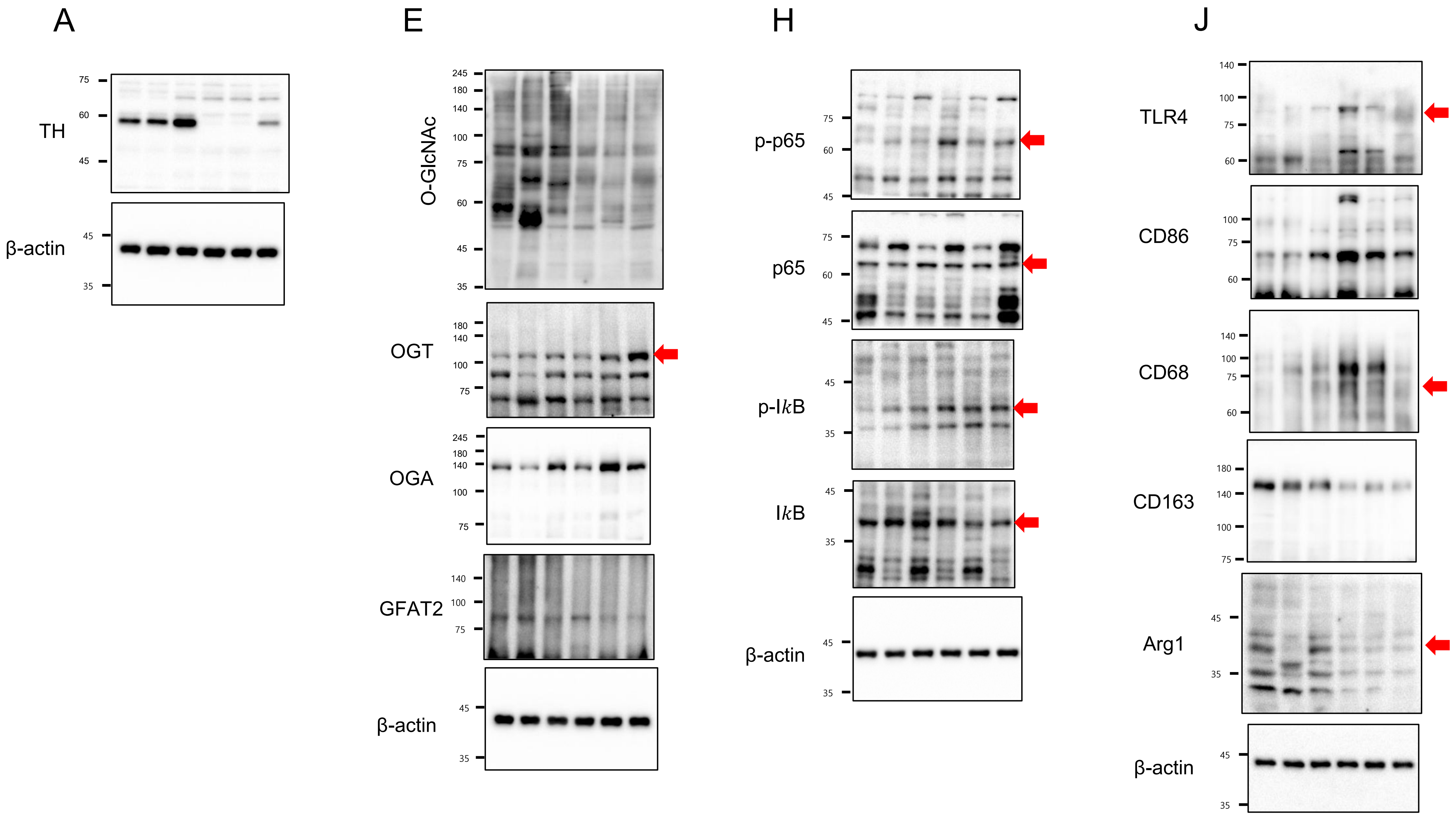
A
TH
β-actin
E
O-GlcNAc
OGT
OGA
GFAT2
β-actin
H
p-p65
p65
p-IkB
IkB
β-actin
J
TLR4
CD86
CD68
CD163
Arg1
β-actin

Figure 1

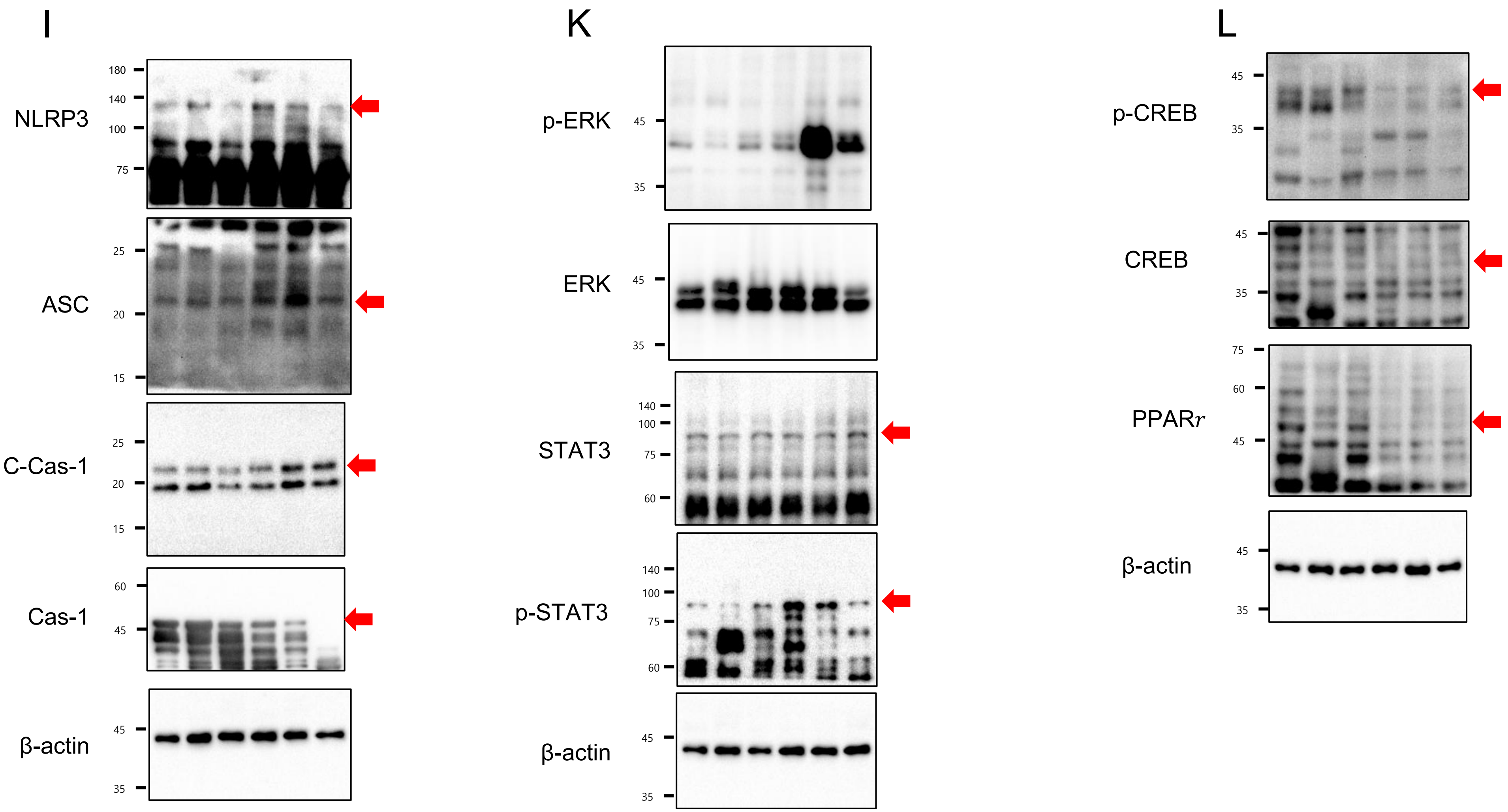
I
NLRP3
180
140
100
75
ASC
25
20
15
C-Cas-1
25
20
15
Cas-1
60
45
β-actin
45
35
K
p-ERK
45
35
ERK
45
35
STAT3
140
100
75
60
p-STAT3
140
100
75
60
β-actin
45
35
L
p-CREB
45
35
CREB
45
35
PPARr
75
60
45
β-actin
45
35

Figure 2

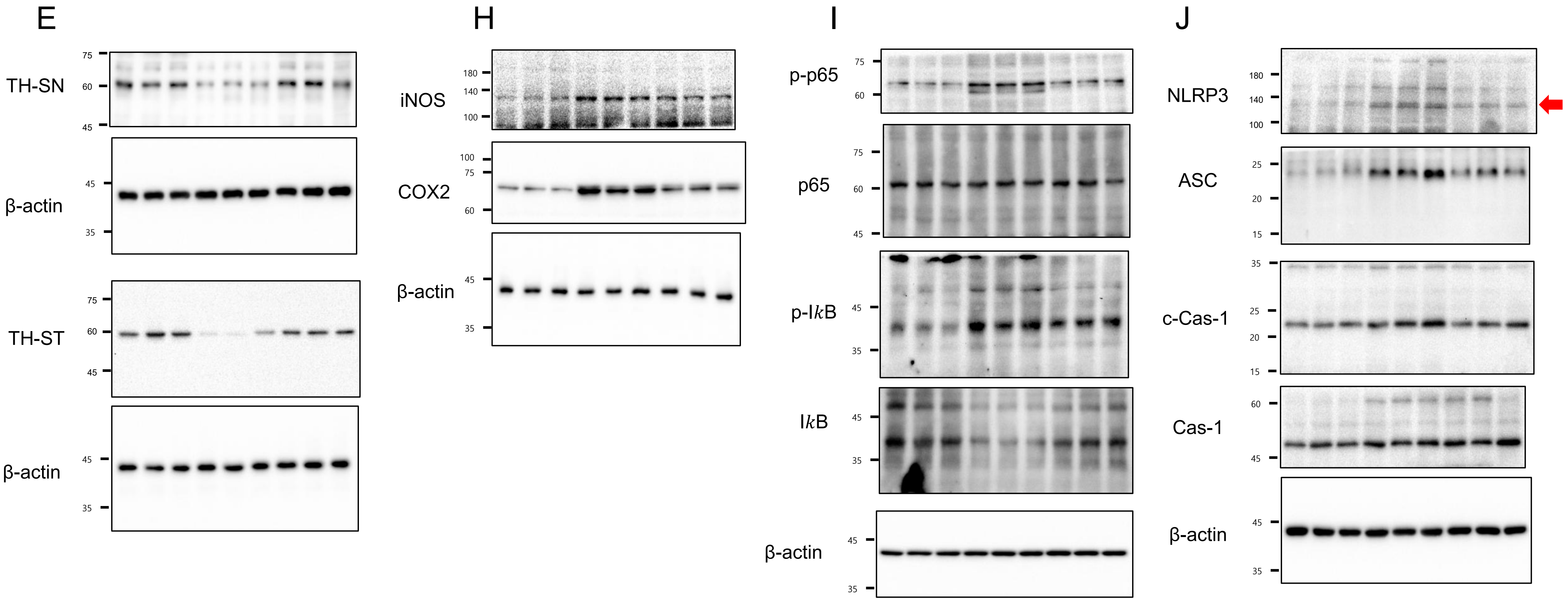
E
TH-SN
β-actin
TH-ST
β-actin
H
iNOS
COX2
β-actin
I
p-p65
p65
p-IkB
IkB
β-actin
J
NLRP3
ASC
c-Cas-1
Cas-1
β-actin

Figure 3

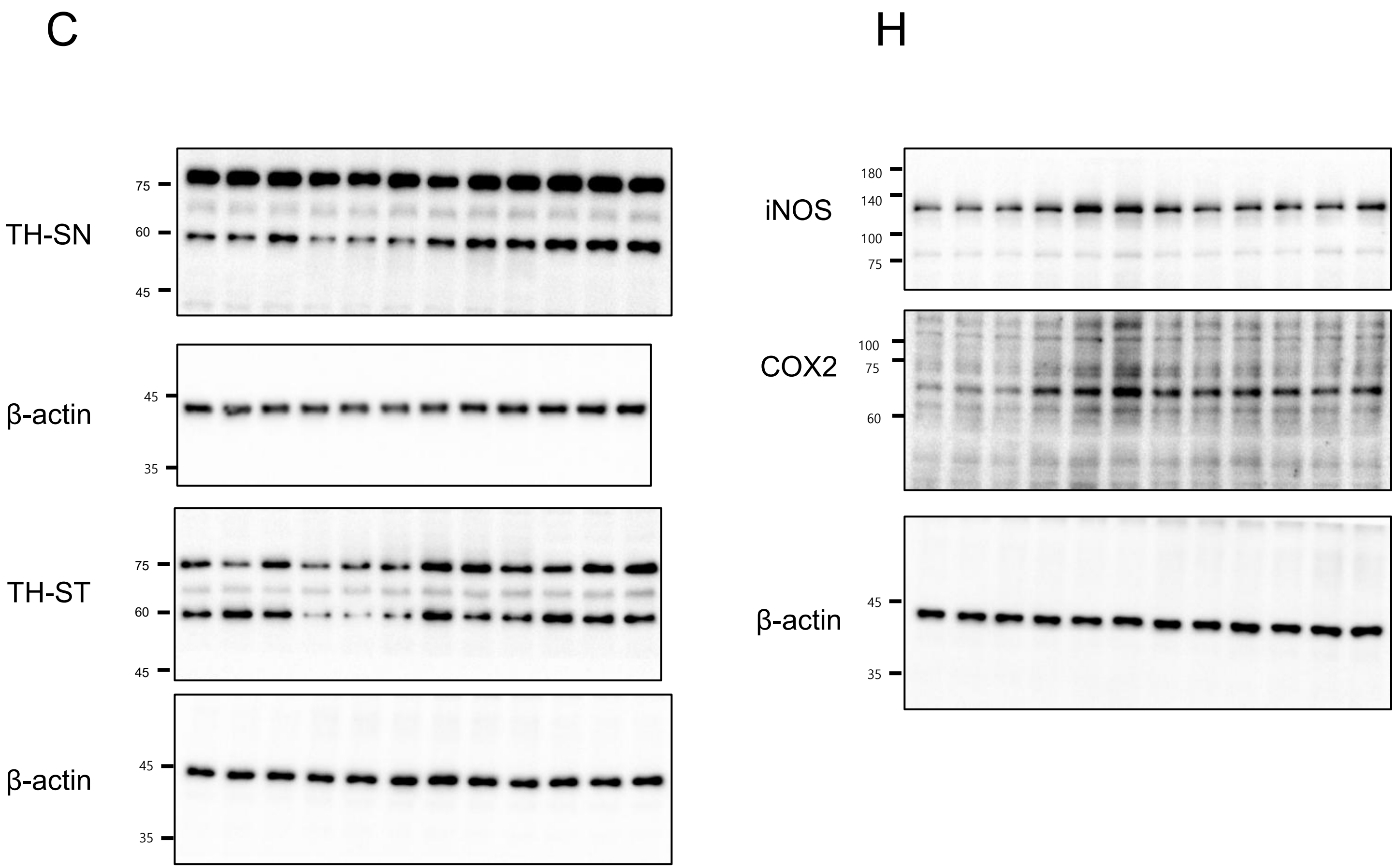
C
H
TH-SN
75
60
45
β-actin
45
35
TH-ST
75
60
45
β-actin
45
35
iNOS
180
140
100
75
COX2
100
75
60
β-actin
45
35

Figure 3

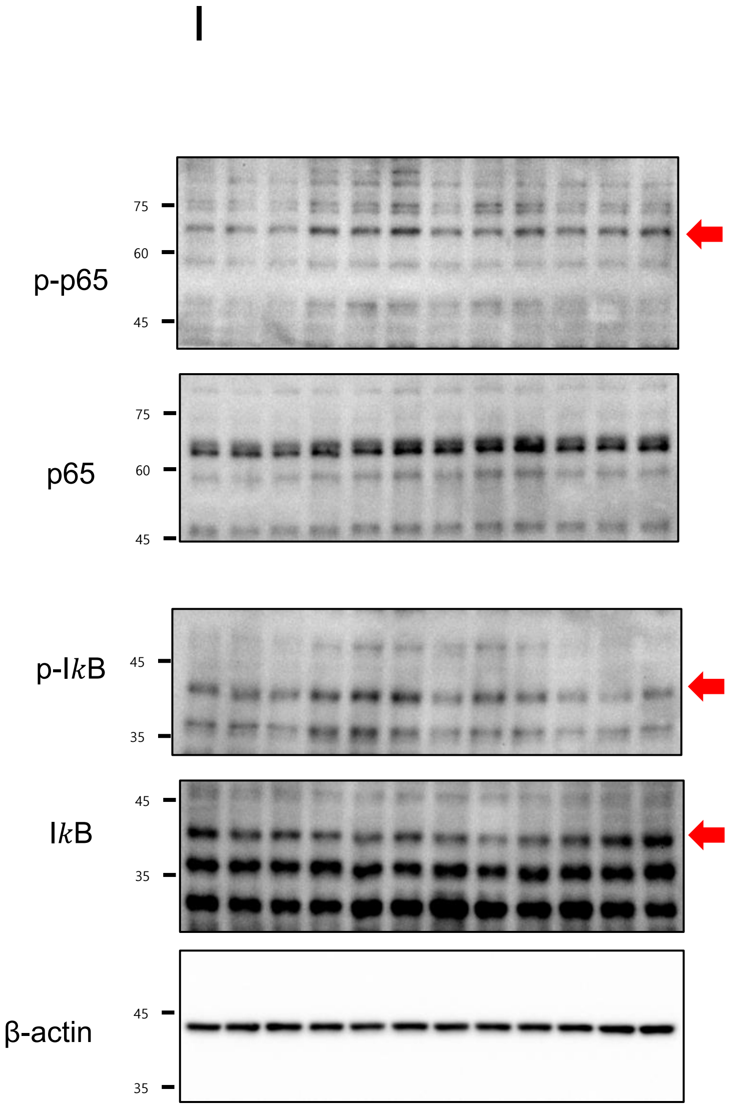
I
p-p65
75
60
45
p65
75
60
45
p-IkB
45
35
IkB
45
35
β-actin
45
35

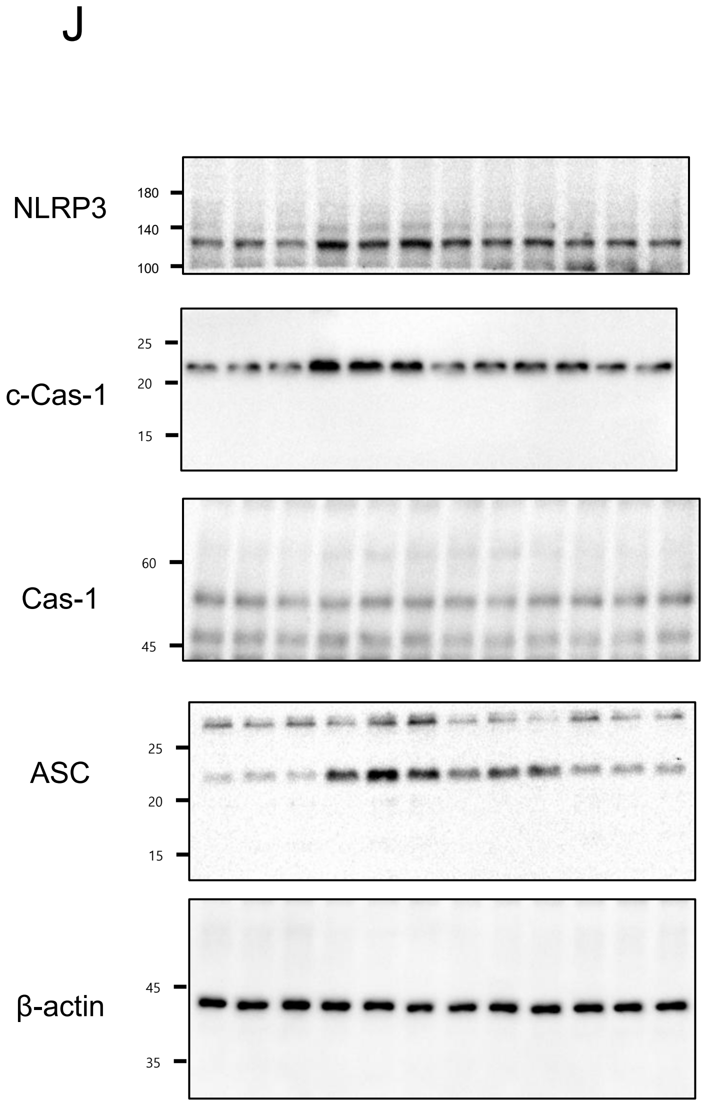
J
NLRP3
180
140
100
c-Cas-1
25
20
15
Cas-1
60
45
ASC
25
20
15
β-actin
45
35

Figure 4

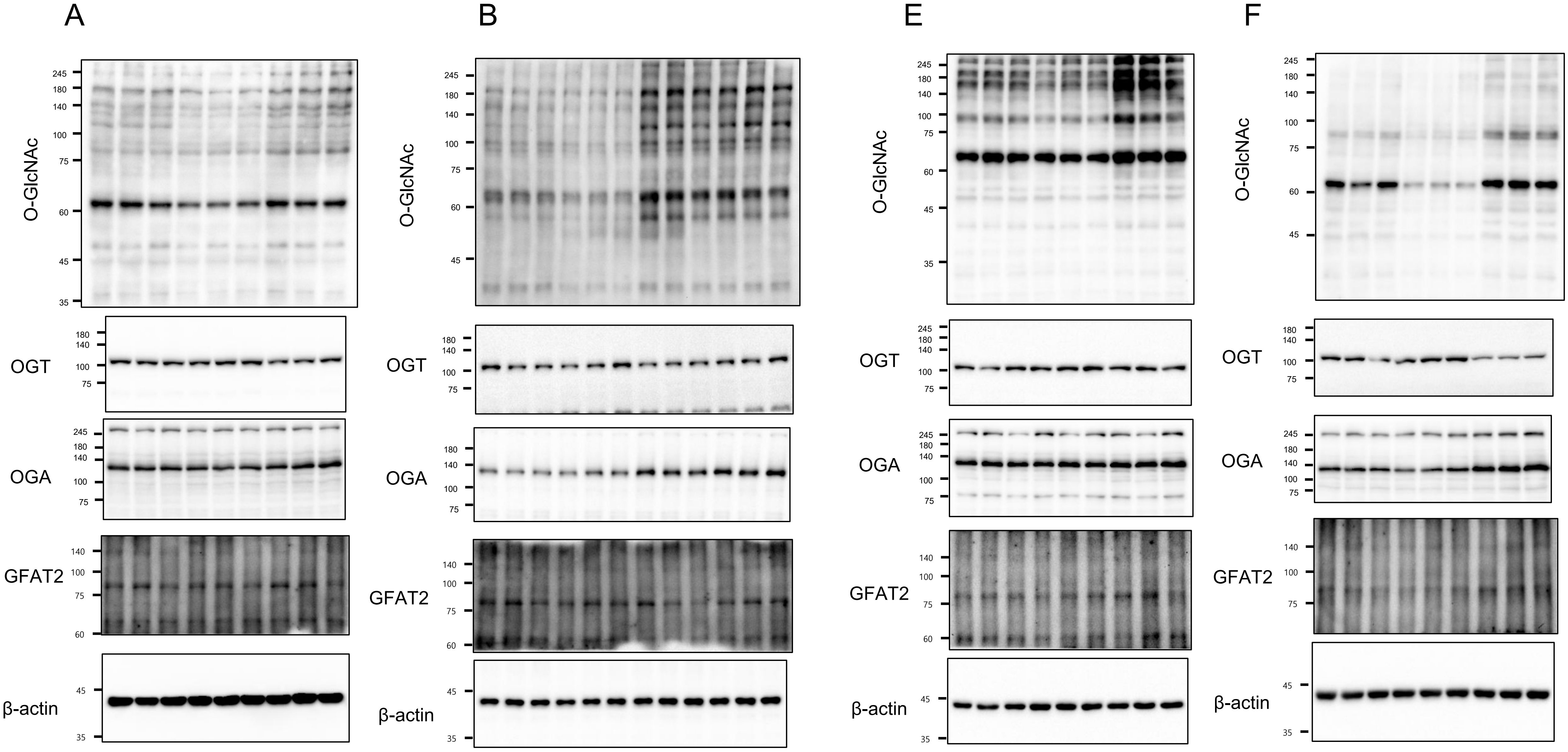

Figure 5

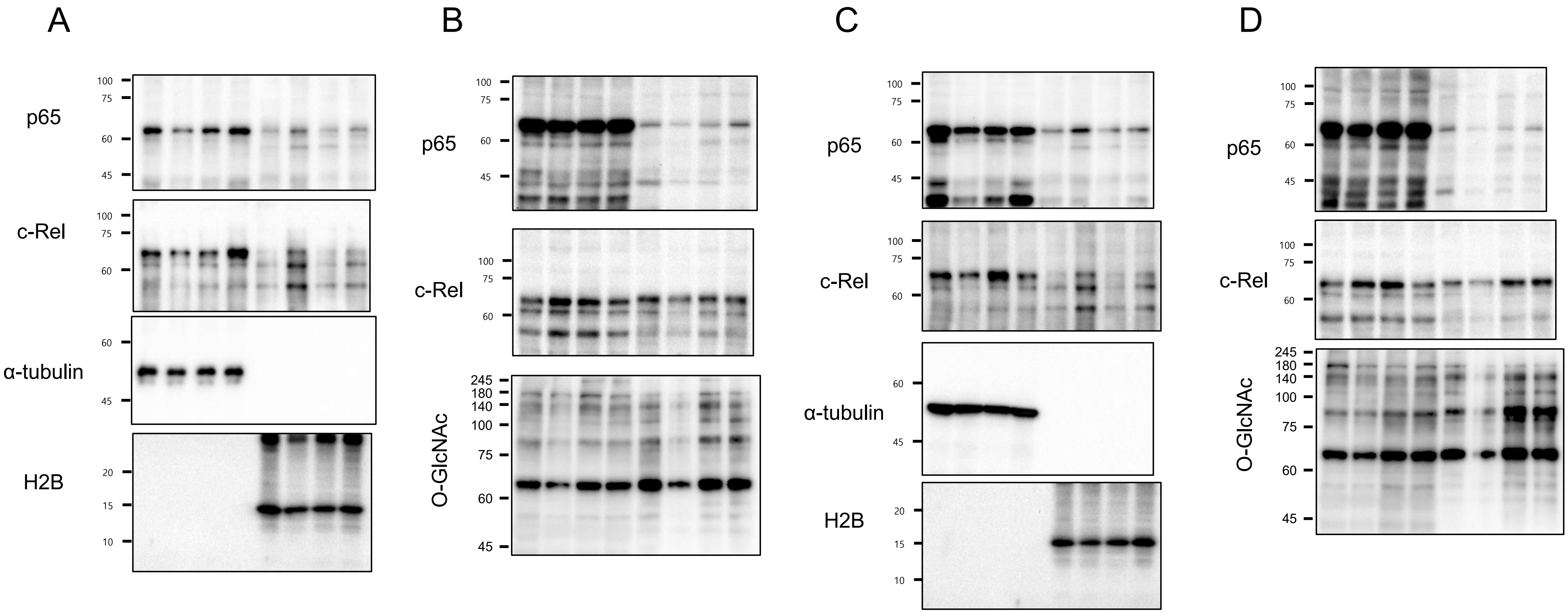
A
p65
100
75
60
45
c-Rel
100
75
60
α-tubulin
60
45
H2B
20
15
10
B
p65
100
75
60
45
c-Rel
100
75
60
O-GlcNAc
245
180
140
100
75
60
45
C
p65
100
75
60
45
c-Rel
100
75
60
α-tubulin
60
45
H2B
20
15
10
D
p65
100
75
60
45
c-Rel
100
75
60
O-GlcNAc
245
180
140
100
75
60
45

Figure 5

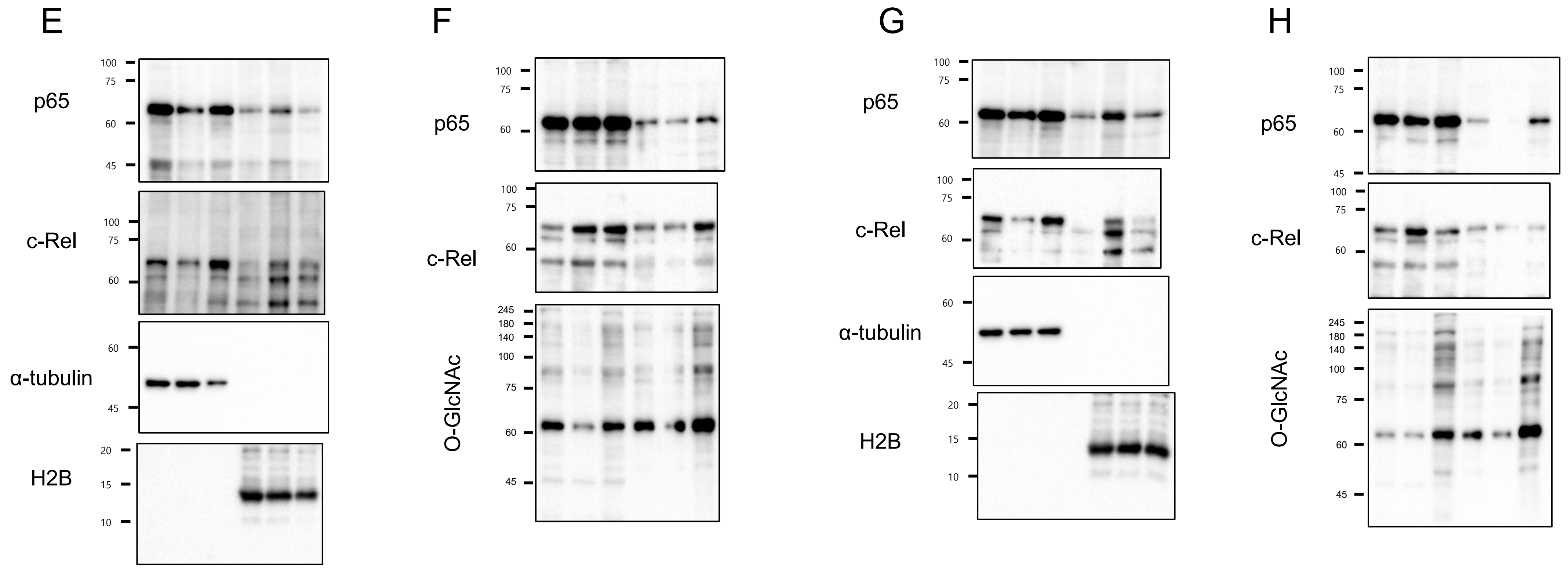
E
F
G
H
p65
c-Rel
α-tubulin
H2B
O-GlcNAc
245
180
140
100
75
60
45
20
15
10

Figure 6

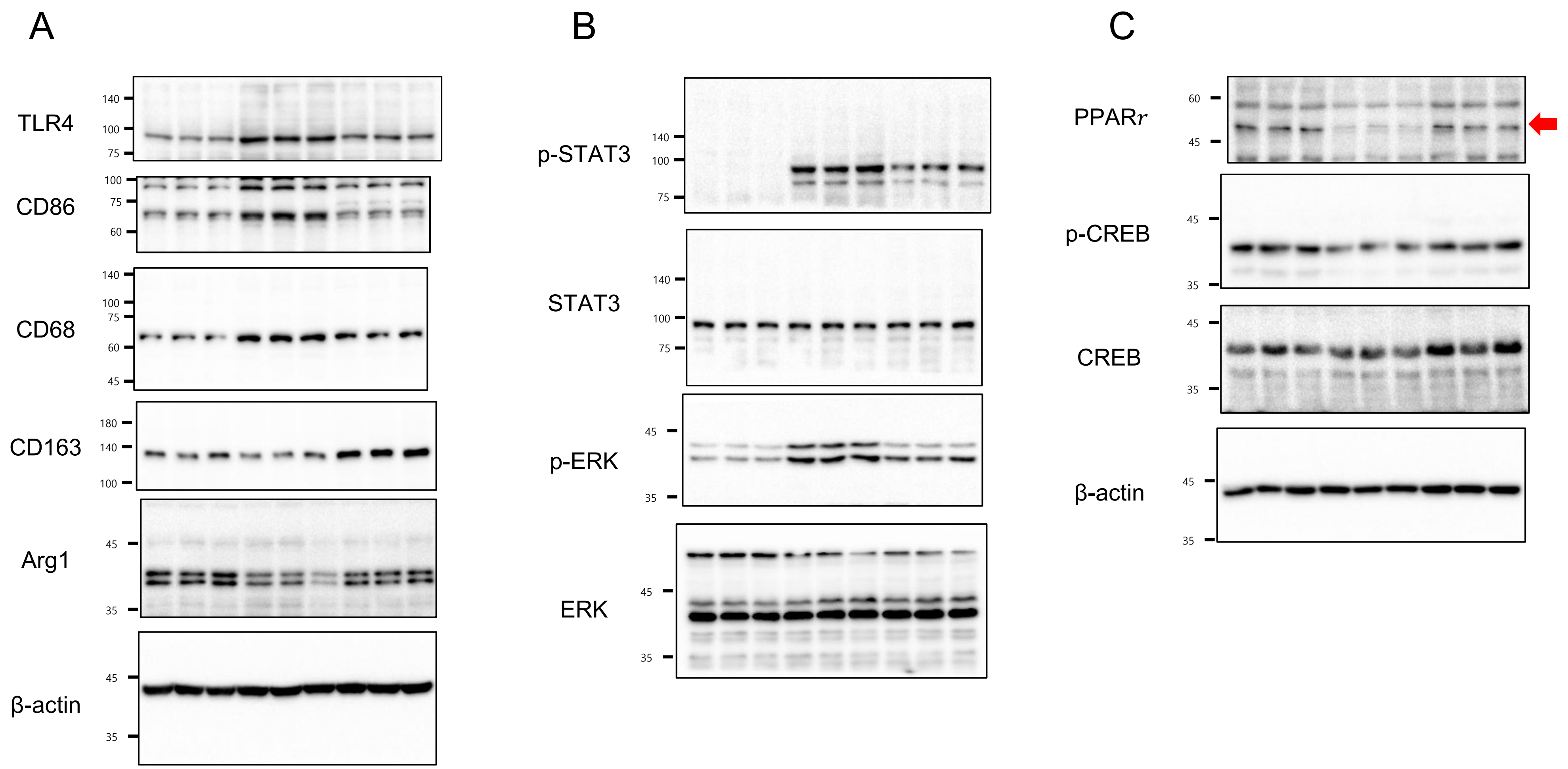
A
TLR4
140
100
75
CD86
100
75
60
CD68
140
100
75
60
45
CD163
180
140
100
Arg1
45
35
β-actin
45
35
B
p-STAT3
140
100
75
STAT3
140
100
75
p-ERK
45
35
ERK
45
35
C
PPARr
60
45
p-CREB
45
35
CREB
45
35
β-actin
45
35

Figure 6

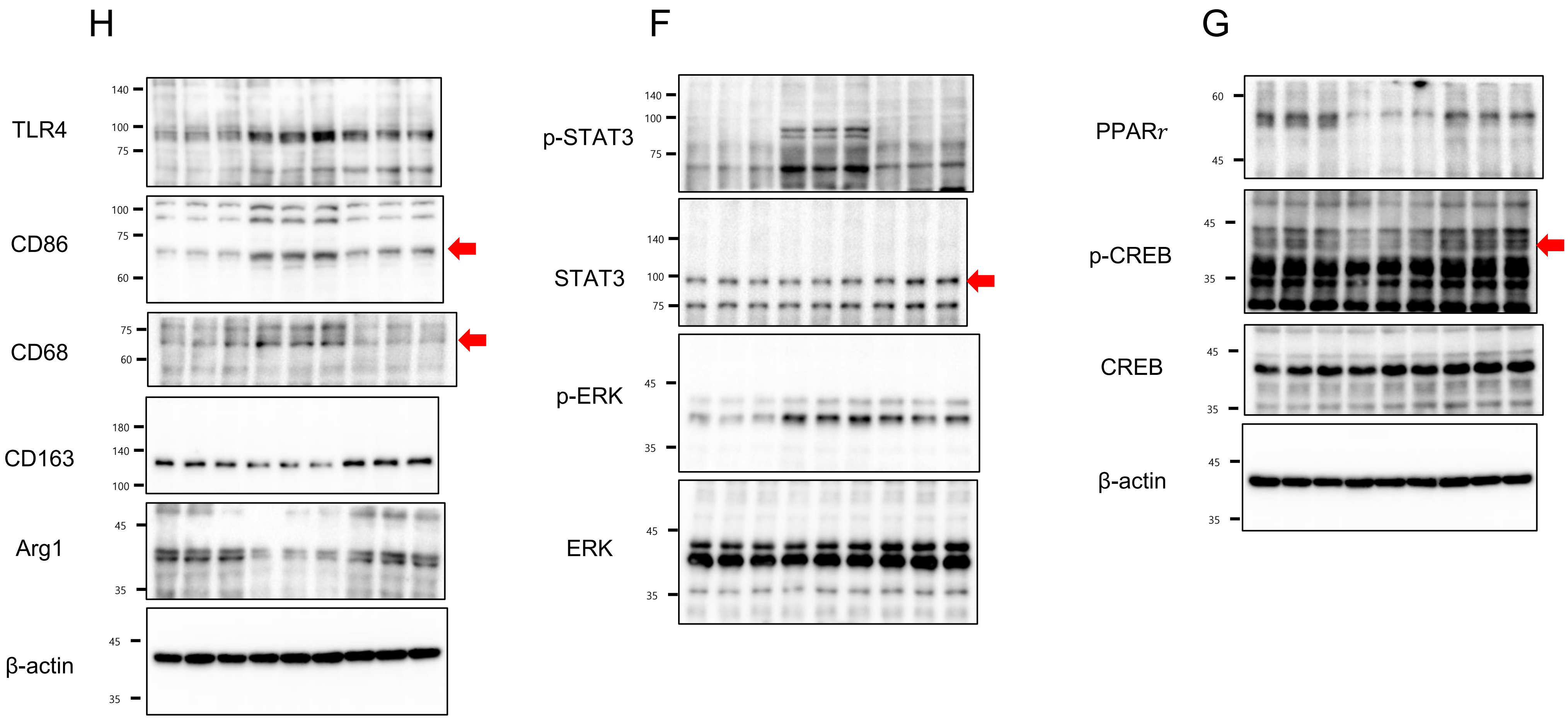
H
F
G
TLR4
140
100
75
CD86
100
75
60
CD68
75
60
CD163
180
140
100
Arg1
45
35
β-actin
45
35
p-STAT3
140
100
75
STAT3
140
100
75
p-ERK
45
35
ERK
45
35
PPARr
60
45
p-CREB
45
35
CREB
45
35
β-actin
45
35

Figure 7

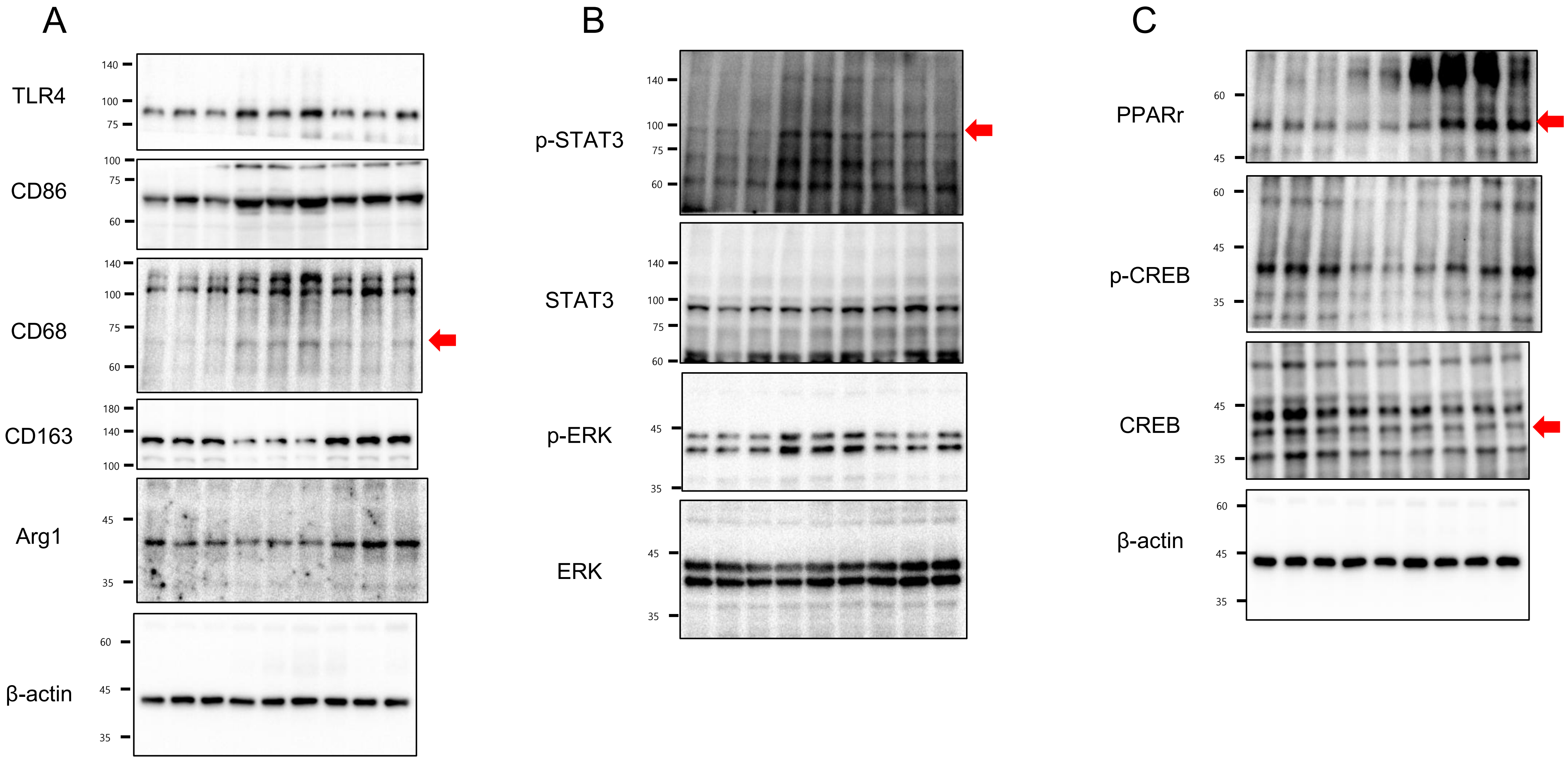
A
TLR4
CD86
CD68
CD163
Arg1
β-actin
140
100
75
60
180
45
35
B
p-STAT3
STAT3
p-ERK
ERK
C
PPARr
p-CREB
CREB
β-actin

Figure 7

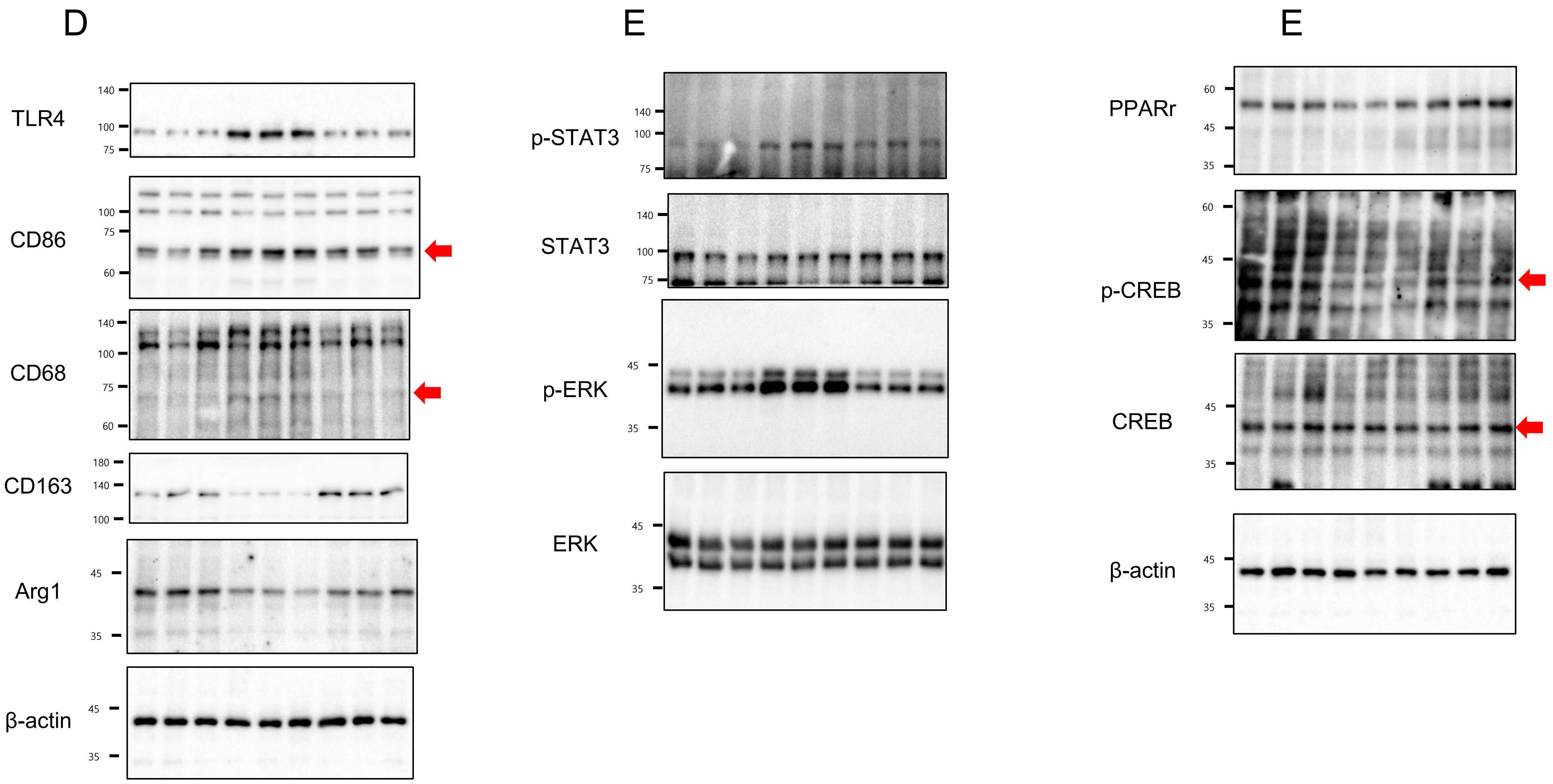
D
TLR4
140
100
75
CD86
100
75
60
CD68
140
100
75
60
CD163
180
140
100
Arg1
45
35
β-actin
45
35
E
p-STAT3
140
100
75
STAT3
140
100
75
p-ERK
45
35
ERK
45
35
E
PPARr
60
45
35
p-CREB
60
45
35
CREB
45
35
β-actin
45
35

Figure 8

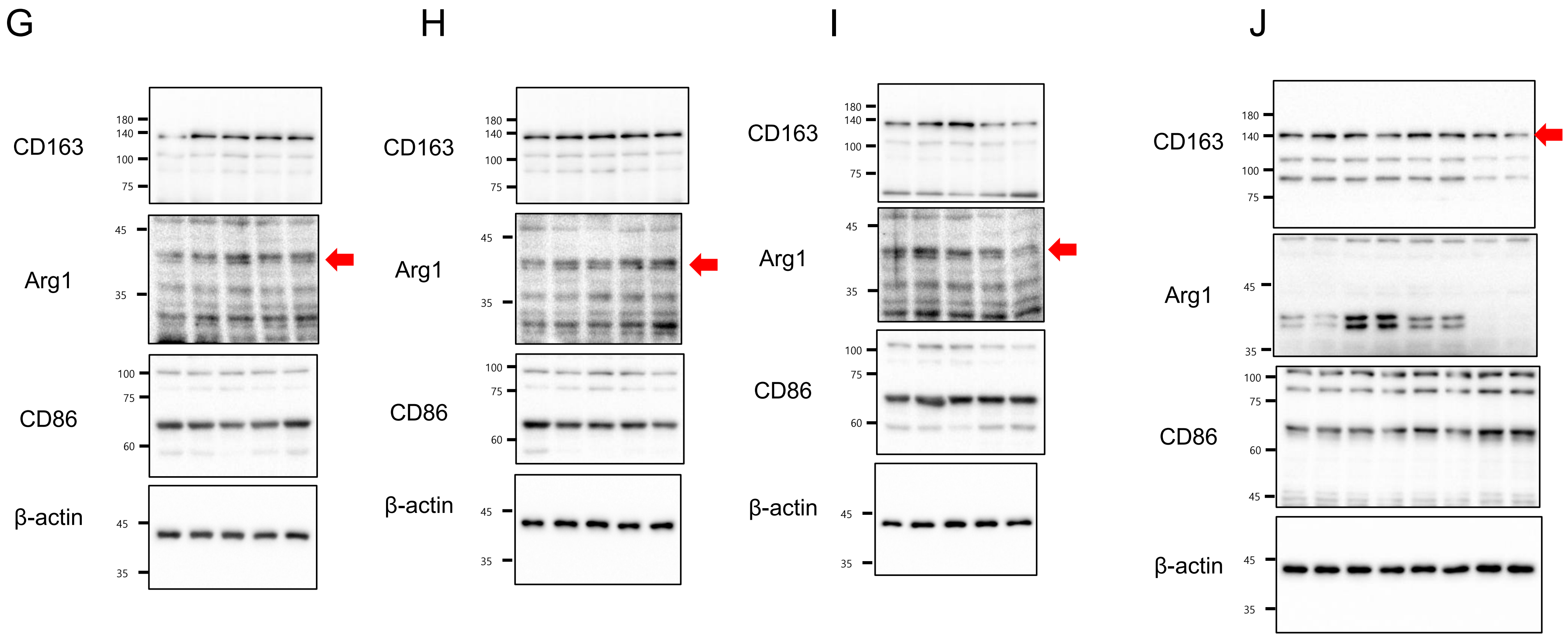
G
CD163
180
140
100
75
Arg1
45
35
CD86
100
75
60
β-actin
45
35
H
CD163
Arg1
CD86
β-actin
I
CD163
Arg1
CD86
β-actin
J
CD163
Arg1
CD86
β-actin

Supplementary Figure 1

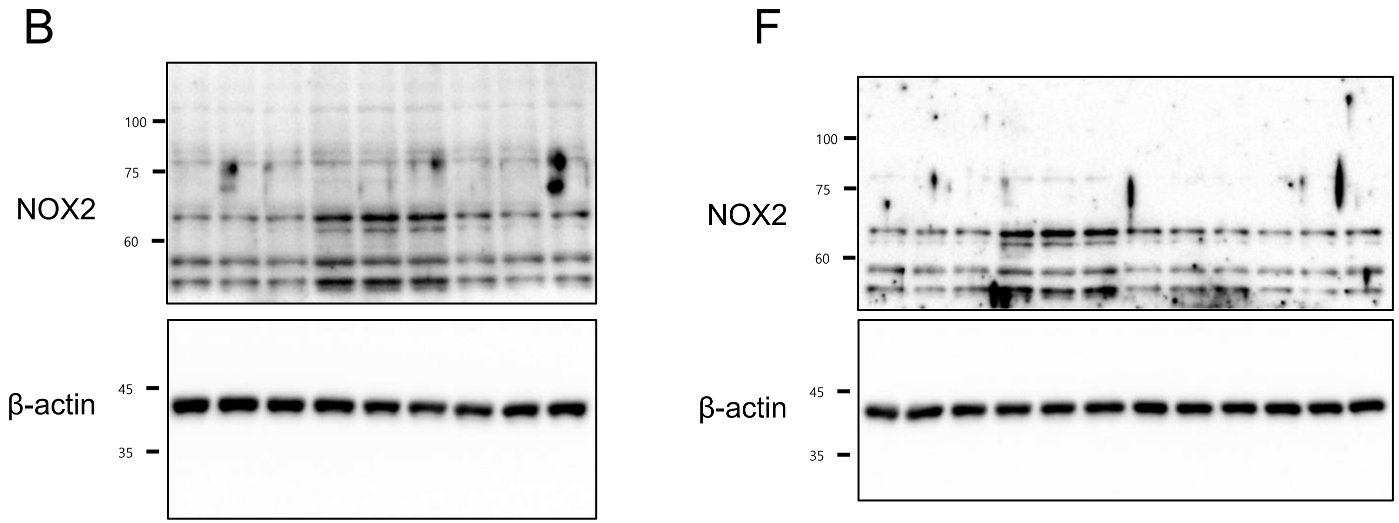

Supplementary Figure 4

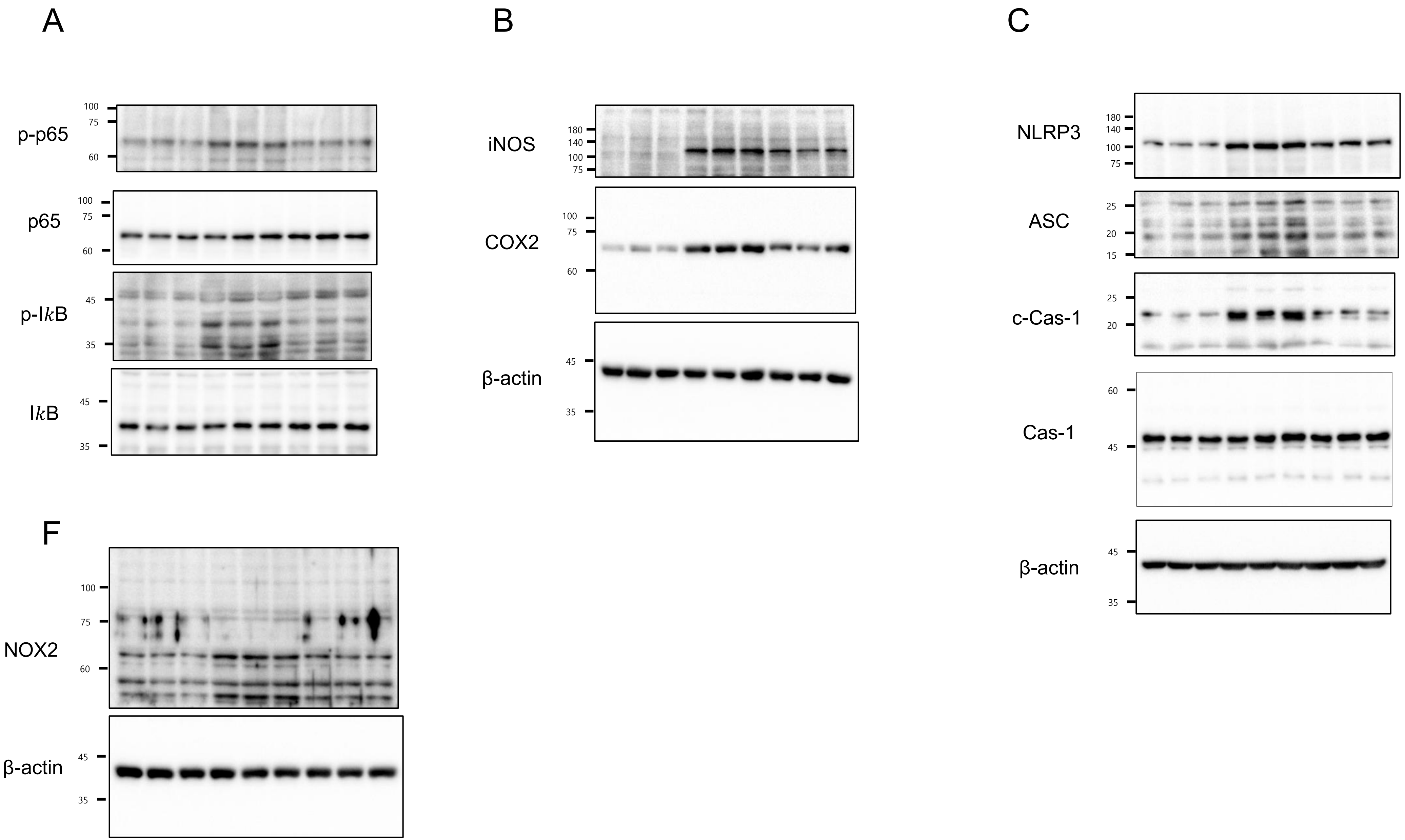

Supplementary Figure 5

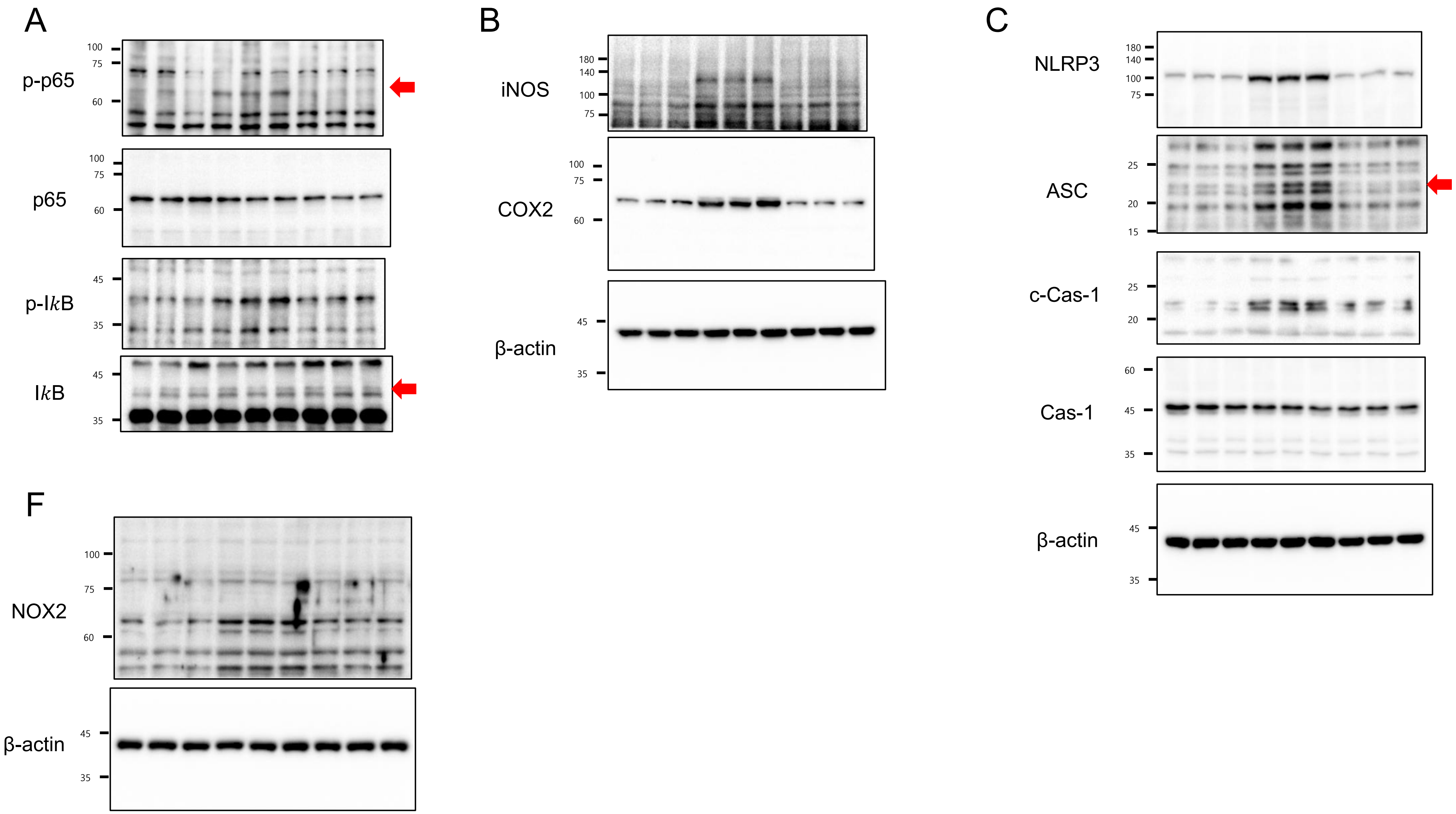

Supplementary Figure 6

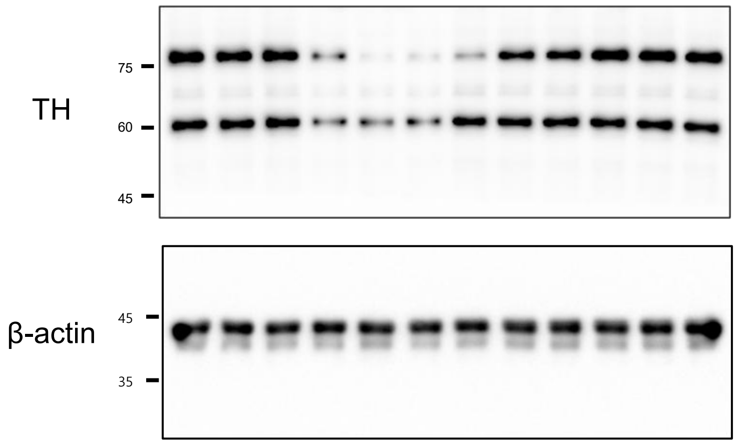

Supplementary Figure 7

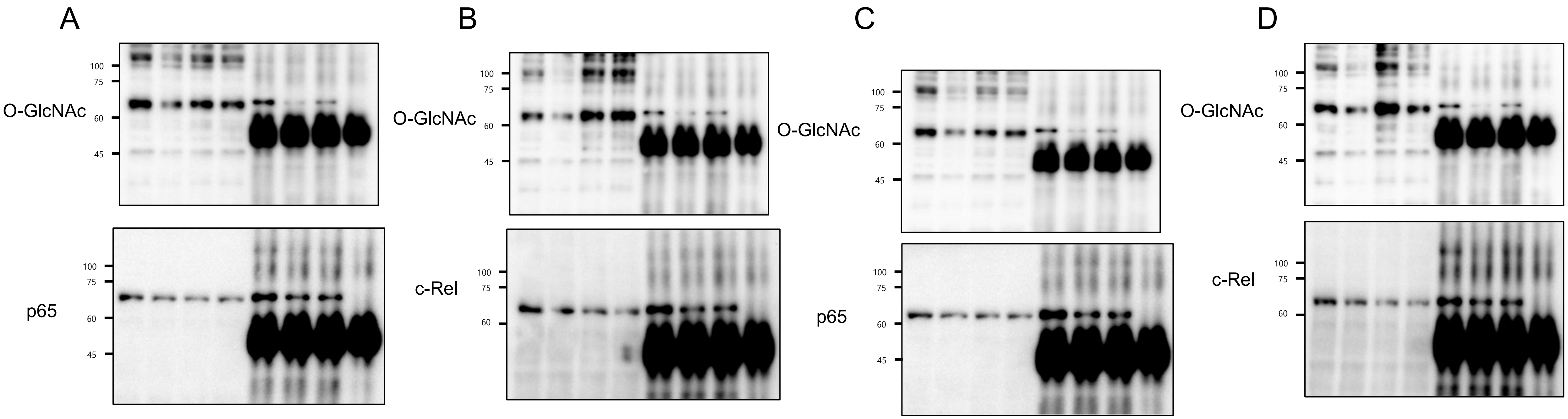

Supplementary Figure 8

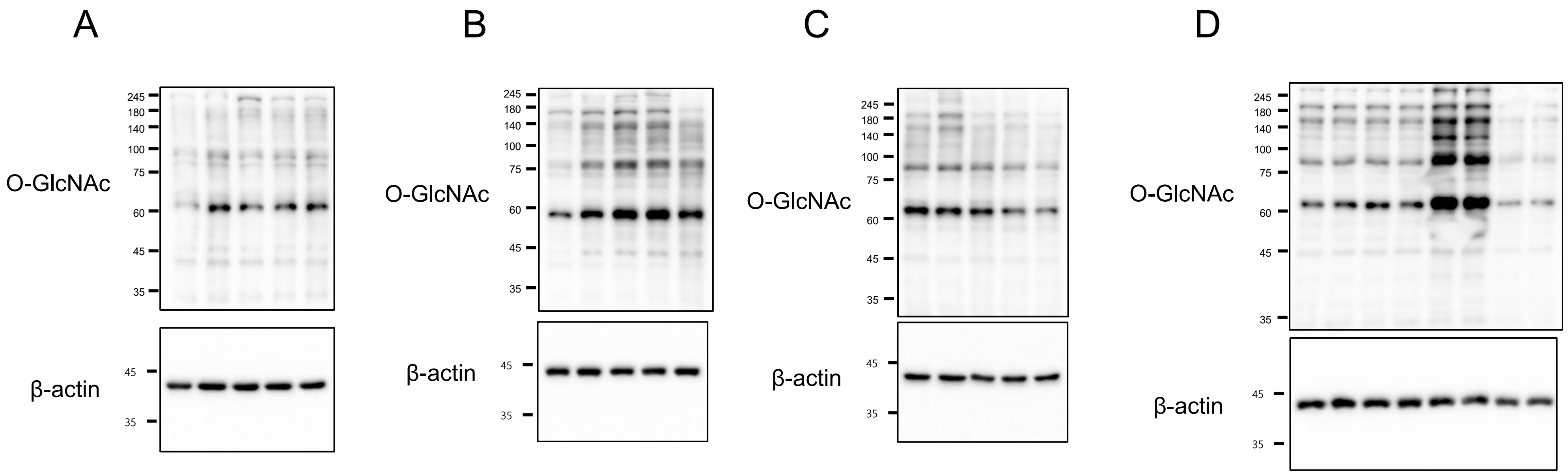

Supplement: Supplementary file 2 — 41531_2026_1319_MOESM2_ESM [file 41531_2026_1319_MOESM2_ESM.pdf]
